# Efficient bidirectional piezo-optomechanical transduction between microwave and optical frequency

**DOI:** 10.1038/s41467-020-14863-3

**Published:** 2020-03-03

**Authors:** Wentao Jiang, Christopher J. Sarabalis, Yanni D. Dahmani, Rishi N. Patel, Felix M. Mayor, Timothy P. McKenna, Raphaël Van Laer, Amir H. Safavi-Naeini

**Affiliations:** Department of Applied Physics and Ginzton Laboratory, Stanford University, 348 Via Pueblo Mall, Stanford, CA 94305 USA

**Keywords:** Nanoscale devices, Nanophotonics and plasmonics, Optomechanics

## Abstract

Efficient interconversion of both classical and quantum information between microwave and optical frequency is an important engineering challenge. The optomechanical approach with gigahertz-frequency mechanical devices has the potential to be extremely efficient due to the large optomechanical response of common materials, and the ability to localize mechanical energy into a micron-scale volume. However, existing demonstrations suffer from some combination of low optical quality factor, low electrical-to-mechanical transduction efficiency, and low optomechanical interaction rate. Here we demonstrate an on-chip piezo-optomechanical transducer that systematically addresses all these challenges to achieve nearly three orders of magnitude improvement in conversion efficiency over previous work. Our modulator demonstrates acousto-optic modulation with ${V}_{\pi }$ = 0.02 V. We show bidirectional conversion efficiency of $1{0}^{-5}$ with 3.3 μW  red-detuned optical pump, and $5.5 \%$ with 323 μW blue-detuned pump. Further study of quantum transduction at millikelvin temperatures is required to understand how the efficiency and added noise are affected by reduced mechanical dissipation, thermal conductivity, and thermal capacity.

## Abstract (web summary)

Current optomechanical implementations of microwave and optical frequency interconversion are lacking in efficiency and interaction strength. The authors design and demonstrate an on-chip piezo-optomechanical solution which overcomes several technical barriers to reach several orders of magnitude improvement in efficiency.

## Introduction

It takes energy to sufficiently change the optical properties of a device or medium to impart information onto an optical field^1,2^. Electro-optic devices are engineered to minimize this energy by using low-loss optical waveguides and resonators that localize the optical field in a small volume, and reduce the amount of energy it takes to set up the electric fields needed for modulation^3,4^. Mechanical vibrations change the local optical properties with less energy than is typically possible via the electro-optic effect in common materials. Whereas voltages corresponding to $\approx \!1{0}^{10}$ microwave photons are typically needed in the most highly optimized electro-optic systems, only $\approx \!1{0}^{4}$ microwave phonons of the same energy are needed in the best optomechanical systems^5^. However, this efficient modulation requires localization of both optical and mechanical energy into a wavelength-scale volume^5^. This complicates electrical driving of this localized mechanical motion and must be addressed by careful co-engineering of a piezo-optomechanical system.

An energy-efficient modulator, whether electro-optic or piezo-optomechanical, also operates as a quantum transducer between microwaves and light where a large optical pump coherently and reversibly couples an optical sideband to microwave-frequency excitations^6–8^. Such transducers may one day enable quantum networks that perform distributed quantum sensing and information processing^9,10^. There are many physical mechanisms that mediate the exchange of microwave and optical photons^11^. Among these, as in the classical case, mechanically mediated conversion offers strong coupling rates and low energy consumption^5,12,13^. Electro-optomechanical conversion using MHz-frequency mechanical membranes^14,15^ to mediate interactions between a Fabry–Pérot cavity and a superconducting microwave resonator has been demonstrated with $47 \%$ efficiency, and 38 added noise photons^16^. Desire for larger conversion rates, lower added noise, and lower heating have motivated investigations of integrated gigahertz-frequency devices which require less optical pump power to operate. Several approaches using aluminum nitride (AlN)^17–20^, silicon^21,22^, gallium arsenide (GaAs)^23–25^, and lithium niobate (LN)^26–28^ have been pursued, but the best end-to-end conversion efficiencies have remained on the order of $1{0}^{-8}$^19^.

Efficient piezo-optomechanical modulation is challenging. The mechanical modes must be highly co-localized with the optical resonances to achieve high optomechanical interaction rates, while maintaining electrical access to the mechanical motion. Optomechanical crystals (OMC) provide a natural way to achieve the former by implementing a simultaneous photonic–phononic crystal to confine both optical and mechanical waves^29^. However, efficient electrical coupling to the micron-scale mechanical resonances of a phononic crystal has only recently been achieved^30,31^. These demonstrations leverage the high piezoelectric coefficient of lithium niobate^32^ and electrodes on or near the resonator to efficiently couple motion to high-impedance superconducting microwave circuits. Such an approach is difficult to integrate with photonic devices due the large optical absorption of metals, which ruins the optical quality factor and destroys the superconductivity.

We overcome the low microwave-to-mechanical efficiency of previously demonstrated lithium niobate piezo-optomechanical crystals^27^ by integrating an interdigitated transducer (IDT) that excites a wavelength-scale mechanical waveguide^33^ to efficiently drive the localized mechanical mode of the OMC. Moreover, the phononic waveguide spatially separates the optical and microwave circuits, an important feature in a cryogenic setting where absorption in metals needs to be minimized. We characterize the device as a classical modulator where an incoming microwave signal at the mechanical mode frequency modulates the optical cavity resonance. In a separate experiment, we characterize the potential of the device as a quantum transducer, by imparting a laser tone red-(blue-)detuned by a mechanical frequency from the optical resonance to introduce an interaction between photons resonant with the cavity and the mechanical motion of the device, which is coupled to the external microwave channel. We demonstrate bidirectional conversion between microwave and optical photons with quantum efficiency up to $1{0}^{-5}$ ($5.5 \%$) using the red-(blue-)detuned optical pump. The integrated piezo-optomechanical transducer is fabricated with $X$-cut thin-film lithium niobate on silicon (LNOS), a material platform demonstrated to be compatible with superconducting circuits and qubits^30,31^—opening a path for integration with quantum sensors and processors.

## Results

### Design

**Fig. 1:**
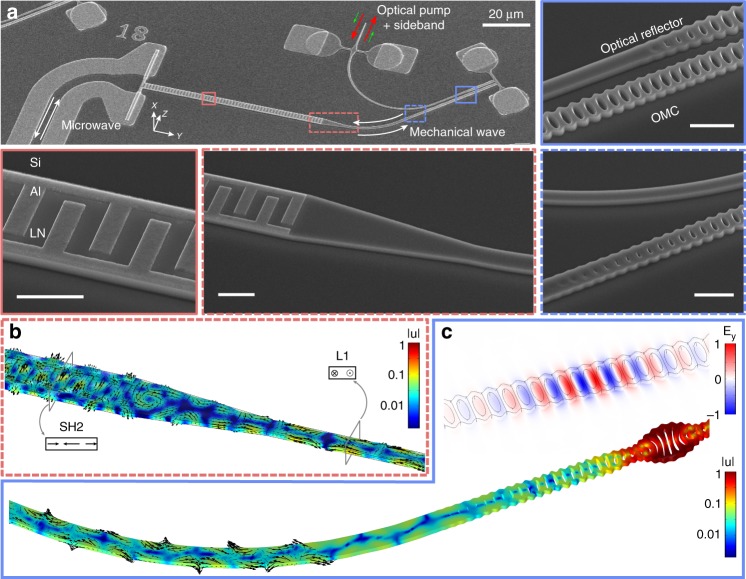
Design of lithium niobate piezo-optomechanical transducers. **a** Scanning electron micrographs (SEM) of one piezo-optomechanical transducer. Zoomed-in SEMs show the conversion region between microwave and mechanics (red) and between mechanics and optics (blue). The tapers between the IDT and the mechanical waveguide (dashed red) and between the optomechanical crystal and the waveguide (dashed blue) are also shown in detail. All scale bars in zoomed-in SEMs are 2 µm. **b** Finite-element simulation of the IDT-to-waveguide taper (normalized displacement). Second-order horizontal shear mode (SH2) in the IDT and first-order longitudinal mode (L1) in the waveguide are indicated. **c** Finite-element simulations of the OMC optical mode (transverse $E$ component) and the leaky mechanical mode (normalized displacement). First-order longitudinal motion in the waveguide can be observed for both the IDT and the leaky OMC mechanical mode.

An incident microwave signal on the IDT is converted to a propagating mechanical wave in the second-order horizontal shear mode (SH2) in the transducer region. The mechanical wave is then scattered by the linear horn into the first-order longitudinal mode (L1) of a 1.3-µm-wide waveguide (Fig. 1b). From finite-element simulation and separate measurement^33^, we determine that $\sim \!0.8 \%$ of the microwave power absorbed by the IDT is converted to mechanical motion in the waveguide, the rest of which is lost to dissipation and clamping. From this fraction, $75 \%$ is in the L1 mode leading to a $0.6 \%$ conversion from microwave input to mechanical power in the L1 mode for a perfectly matched IDT. Phonons propagate down the waveguide and are scattered into the guided mode of the OMC by smoothly ramping on the OMC’s periodic modulation, where the L1 band of the waveguide transforms to the L1 band of the OMC mirror cell (Supplementary Fig. 1). Scattering of mechanical waves from the waveguide into the localized mechanical mode can be induced by breaking a symmetry of the structure. This occurs automatically in our device, in contrast to previous work on silicon OMCs^34^, which required patterns that explicitly broke symmetry to induce scattering. This is because the lithium niobate material lacks crystal symmetry along the reflection plane of the OMC geometry^27^. We simulate a decay rate ${\gamma }_{{\rm{e}}}/2\pi \approx$ 460 kHz using COMSOL^35^, $64 \%$ of which is converted to the L1 mode (Fig. 1c). The IDT pitch $a\approx$ 2.68 µm is chosen to match the frequencies of the IDT and OMC. Since LN is anisotropic, both the IDT and the OMC have optimal orientations where the piezoelectric and photoelastic effects are, respectively, maximized. A curved waveguide with a bending radius of 30 µm connects the two components.

Optical photons are injected into an on-chip edge coupler via a lensed fiber with coupling efficiency ${\eta }_{{\rm{oc}}} \sim 65 \%$. This waveguide is brought in the near-field of the OMC to allow coupling of light fields in and out of the optical resonance. The fabrication process is described in refs. ^27,33^. The scanning electron micrographs (SEM) in Fig. 1 were taken before the final masked release step.

### Device characterization

**Fig. 2:**
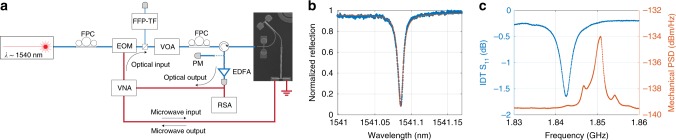
Measurement setup and separate characterizations of the IDT and the OMC. **a** Schematic drawing of the measurement setup. The optical sideband input is generated by a microwave tone from the vector network analyzer (VNA) to the electro-optic modulator (EOM). Optical readout of the sideband is generated by a high-speed photodetector, and is sent back to the VNA. Microwave input and output of the device is directly actuated by and measured from the IDT. **b** Optical resonance of the optomechanical crystal. **c** IDT response (blue) and OMC mechanical mode thermal spectroscopy (red). VOA variable optical attenuator, FPC fiber polarization controller, EDFA erbium-doped fiber amplifier, RSA real-time spectrum analyzer, PM power meter, FFP-TF fiber Fabry–Pérot tunable filter.

Figure 2a shows the measurement setup used for this work. We use a vector network analyzer (VNA) to generate and readout signals to and from the IDT (microwave input and output). A commercial electro-optic modulator (EOM) is driven by the VNA to generate the optical sideband input (optical input). The light reflected from the device containing the pump and sideband is amplified and collected by a high-speed detector, which downconverts the optical sideband to a microwave signal received by the VNA (optical output). We measure all four scattering parameters of this two-port setup at room temperature. All ports are calibrated to de-embed the scattering parameters of the device.

We begin by characterizing the OMC and the IDT. A Lorentzian response is observed when we scan a tunable telecom laser with frequency ${\omega }_{{\rm{p}}}$ across the optical resonance of the OMC at frequency ${\omega }_{{\rm{c}}}$ (Fig. 2b). Combined with the optical sideband response (Supplementary Note 3), we extract a total optical linewidth $\kappa /2\pi =1210\pm 40\,{\rm{MHz}}$ and an external coupling rate ${\kappa }_{{\rm{e}}}/2\pi =800\pm 30\,{\rm{MHz}}$, corresponding to a loaded quality factor $Q=1.6\times 1{0}^{5}$ and an intrinsic quality factor ${Q}_{{\rm{i}}}=4.7\times 1{0}^{5}$. The IDT response is measured with a calibrated microwave probe^33^ and is shown in Fig. 2c. We achieve a peak conductance of $1.9\,{\rm{mS}}$ and a bandwidth of ${B}_{{\rm{IDT}}}=3.36\,{\rm{MHz}}$ with direct coupling to a 50 $\Omega$ transmission line. Lastly, we characterize the mechanical mode and optomechanical coupling by thermal spectroscopy and optomechanical back action^8,27^. A typical thermal mechanical power spectral density (PSD) is plotted in Fig. 2c in red. The fundamental mechanical breathing mode is at ${\omega }_{{\rm{m}}}/2\pi =1.85\,{\rm{GHz}}$. Satellite peaks are visible and indicate the coupling between the local breathing mode and mechanical waveguide modes. We measure a backaction-free mechanical linewidth $\gamma /2\pi =1.93\,{\rm{MHz}}$ and an optomechanical coupling rate ${g}_{0}/2\pi =70\,{\rm{kHz}}$ via the optomechanical backaction (Supplementary Note 4). We note that both $\gamma$ and ${g}_{0}$ differ significantly from the values measured in ref. ^27^ with identical OMC geometry and orientation. We attribute these deviations to hybridization of the local breathing mode with the waveguide modes.

### Efficient acousto-optic modulation

To demonstrate acousto-optic modulation, we drive the mechanical mode of the OMC with a microwave tone at frequency ${\omega }_{\upmu } \sim {\omega }_{{\mathrm{m}}}$ and measure the reflection spectrum of the optical mode. The coherent mechanical motion induces a varying optical cavity frequency

1 $$\widetilde{\omega_{\mathrm{c}}}={\omega }_{{\rm{c}}}+\widetilde{\Delta {\omega }_{{\rm{c}}}}={\omega }_{{\rm{c}}}+2{g}_{0}\sqrt{{n}_{{\rm{phon}}}}\cos {\omega }_{\upmu }t,$$

where ${n}_{{\rm{phon}}}$ is the driven intracavity phonon number proportional to the input microwave power ${P}_{\upmu }$. This phase modulation splits the optical cavity spectrum into sidebands at ${\omega }_{{\rm{c}}}\pm k{\omega }_{\upmu }$ with integer sideband index $k$ and relative strength determined by $k$ and the modulation index $h\equiv 2{g}_{0}\sqrt{{n}_{{\rm{phon}}}}/{\omega }_{\upmu }$ (Supplementary Note 5). We measure the cavity reflection spectrum for different microwave powers and frequencies.

**Fig. 3:**
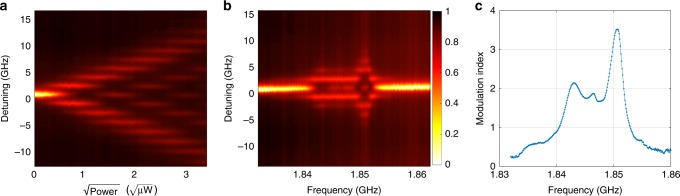
Efficient acousto-optic modulation. **a**, **b** A microwave signal sent to the IDT modulates the optical cavity frequency. The reflection spectrum of the optical cavity is recorded for different microwave power (**a**) and frequency (**b**). Color bar: normalized reflection. **c** Modulation index for different microwave drive frequency, extracted from (**b**).

Figure 3a shows the reflection spectrum versus cavity-laser detuning $\Delta ={\omega }_{{\rm{c}}}-{\omega }_{{\rm{p}}}$ and microwave power ${P}_{\upmu }$. The phase modulation sidebands are clearly resolved. We empirically confirm the expected proportionality between the square root of the microwave power and the modulation index $h$. We show the measured spectrum with fixed power ${P}_{\upmu }$ = 7.24 µW at different frequencies in Fig. 3b, and extract the modulation index versus frequency in Fig. 3c by fitting the spectrum (Supplementary Note 5). The modulation index peaks at the mechanical mode frequency ${\omega }_{\upmu }={\omega }_{{\rm{m}}}$, where the microwave power required to achieve modulation index $h=1$ is as low as ${P}_{\upmu }$ = 0.58 µW. The complex shape of the excitation spectrum in Fig. 3c is due to the mismatch of the IDT and mechanical mode frequencies (see Fig. 2c). The lower local maxima match the IDT response and standing wave modes of the mechanical waveguide. The mismatch between the local mechanical mode frequency and the IDT frequency lowers the peak modulation, but increases the $3\,{\rm{dB}}$ bandwidth to $B \sim 10\,{\rm{MHz}}$. Combining the acousto-optic modulation measurement with ${g}_{0}$ and $\gamma$, we deduce a decay rate from the mechanical mode to the ${Z}_{0}=50\ \Omega$ microwave transmission line of ${\gamma }_{\upmu }/2\pi =2.2\,{\rm{kHz}}$ and the corresponding microwave-to-mechanical conversion efficiency ${\eta }_{{\rm{m}}}\equiv {\gamma }_{\upmu }/\gamma =0.11 \%$ (Supplementary Note 5).

An important figure of merit for a classical modulator is the amount of driving energy required to encode one bit of information on an optical field^3^. This generally requires consideration of multiple aspects of a communication system. We consider a simplified thought experiment, where we begin with the optical resonator prepared initially in a coherent state $| {\alpha }_{0}{|}^{2}=1$ and ask how well can the output optical field $\left|{\Psi }_{k}\right\rangle$ be distinguished when the modulator is driven to encode $k=0$ or $k=1$. In the Supplementary Note 6, we use the Helstrom–Holevo bound^36^ to calculate the driving energy required to realize a bit-error-rate of $10 \%$ for different system parameters $({g}_{0},{\eta }_{{\rm{m}}},\kappa ,{\omega }_{{\rm{m}}})$ and find two limiting cases:

2 $${E}_{{\rm{bit}}}=\frac{\hslash {\omega }_{{\rm{m}}}}{16{\eta }_{{\rm{m}}}}{\left(\frac{\kappa }{{g}_{0}}\right)}^{2}\,\,{\rm{for}}\,\kappa \gg {\omega }_{{\rm{m}}},\,\,{\rm{and}}$$

3 $${E}_{{\rm{bit}}}=\frac{\hslash {\omega }_{{\rm{m}}}}{8{\eta }_{{\rm{m}}}}{\left(\frac{{\omega }_{{\rm{m}}}}{{g}_{0}}\right)}^{2}\,\,{\rm{for}}\,\kappa \ll {\omega }_{{\rm{m}}}.$$

In our sideband-resolved system ($\kappa \, < \, {\omega }_{{\rm{m}}}$), the second equation leads to an energy-per-bit of 97 fJ. Notice that a modulation index of $h=\pi$ is achieved with ${V}_{\pi }$ = 24 mV, requiring and RF power ${P}_{\pi }={V}_{\pi }^{2}/2{Z}_{0}$, which can also be used to estimate the energy-per-bit ${P}_{\pi }/(2\pi B) \sim$ 100 fJ.

### Bidirectional conversion between microwave and optical frequency

We demonstrate microwave-to-optical conversion by pumping the optical mode with detuning $\Delta =\pm \!{\omega }_{{\rm{m}}}$ using different pump powers and measuring the microwave-to-optical scattering parameter. The total conversion efficiency is defined as the ratio between input microwave photon flux before the IDT and output sideband photon flux after the light being collected by the lensed fiber. At $\Delta =\pm \!{\omega }_{{\rm{m}}}$, the efficiency is given by

4 $${\eta }_{{\rm{total}}}={\eta }_{{\rm{oc}}}{\eta }_{{\rm{o}}}{\eta }_{{\rm{m}}}\frac{4C}{{(1\pm C)}^{2}},$$

where ${\eta }_{{\rm{oc}}}=(65.2\pm 0.4) \%$ is the fiber-to-chip optical coupling efficiency and ${\eta }_{{\rm{o}}}\equiv {\kappa }_{{\rm{e}}}/\kappa =(66\pm 1) \%$ is the external optical coupling efficiency. $C={C}_{0}{n}_{{\rm{c}}}$ is the optomechanical cooperativity, ${n}_{{\rm{c}}}$ is the intracavity photon number, and ${C}_{0}\equiv 4{g}_{0}^{2}/(\kappa \gamma )$ is the single-photon cooperativity.

For a red-detuned pump, the linearized optomechanical interaction gives a beam-splitter interaction Hamiltonian for a sideband-resolved system^8^. Ideal internal quantum transduction can be achieved in principle at the matching condition $C=1$, where the maximal efficiency equals the total external coupling efficiency ${\eta }_{{\rm{total}}}={\eta }_{{\rm{e}}}\equiv {\eta }_{{\rm{oc}}}{\eta }_{{\rm{o}}}{\eta }_{{\rm{m}}}$. Alternatively, a blue-detuned optical pump leads to a two-mode squeezing Hamiltonian that could be utilized to generate entanglement between optical and microwave photons, required for long-range quantum communication^37,38^. It also gives rise to phase-conjugating amplification of input optical sideband photons or input microwave photons with added noise^39^. The internal gain ${G}_{{\rm{int}}}\equiv 4C/{(1-C)}^{2}$ monotonically increases with optomechanical cooperativity $C$ until the phonon lasing condition $C\ge 1$, where ${G}_{{\rm{int}}}\to \infty$ and the system no longer operates in the linear regime. In our demonstration, we reached ${G}_{{\rm{int}}}\,{> }\, {1}$ compensating for some, but not all of the external coupling losses ${\eta }_{{\rm{e}}}$. We refer to the scattering parameter measurement under blue-detuned pump as “efficiency” instead of “gain” with the understanding that this is not a quantum-state-conversion process.

**Fig. 4:**
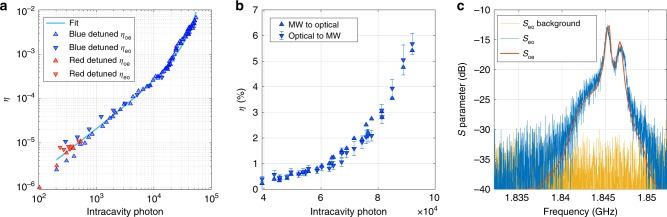
Bidirectional microwave-optical conversion. **a** Conversion efficiency measured with red and blue-detuned pump laser. Upward (downward) triangles represent the microwave-to-optical (optical-to-microwave) conversion efficiencies, where the frequency of the photon is upconverted (downconverted). The optical-to-microwave efficiency ${\eta }_{{\rm{eo}}}$ is measured with a different setup. **b** Conversion efficiency measured with relatively high power blue-detuned pump laser using same measurement setup for ${\eta }_{{\rm{oe}}}$ and ${\eta }_{{\rm{eo}}}$ (Fig. 2a). Error bars indicate one standard deviation. **c** Bidirectional scattering parameter measurement. The maximal $| S{|}^{2}$ is shifted to the calibrated efficiency. The ${S}_{{\rm{eo}}}$ background is measured by sending the VNA output to a 50 $\Omega$ load instead of the EOM.

We calibrate the optical detection gain using a second calibration laser (see Supplementary Note 7 and also ref. ^40^ for detailed description). The calibrated total microwave-to-optical conversion efficiency ${\eta }_{{\rm{oe}}}$ is shown in Fig. 4a for different intracavity photon numbers. Measurements with a red-detuned pump are limited by the thermo-optical instability^27^ to intracavity photon ${n}_{{\rm{c}}}\,{\lesssim}\,{500}$ and a resulting total efficiency ${\eta }_{{\rm{oe}}} \sim 1{0}^{-5}$. We measure the efficiency with a blue-detuned pump up to ${n}_{{\rm{c}}} \sim 4\times 1{0}^{4}$ and fit to Eq. (4) with two fitting parameters ${\eta }_{{\rm{e}}}$ and ${C}_{0}$. The fit curve is plotted in Fig. 4a in cyan, showing good agreement with measured efficiencies across more than three orders of magnitude. We extract a single-photon cooperativity ${C}_{0}=1.2\times 1{0}^{-5}$ and a total external coupling efficiency ${\eta }_{{\rm{e}}}=4.24\times 1{0}^{-4}$. The deviation of measured efficiencies from the fit at high ${n}_{{\rm{c}}}$ can be attributed to thermally induced redshifts of the mechanical mode (Supplementary Note 10). We also calculate ${C}_{0}=8.4\times 1{0}^{-6}$ from independent measurement of ${g}_{0}$, $\kappa$ and $\gamma$. The ${g}_{0}$ obtained from blue-detuned backaction measurement is possibly underestimating the actual ${g}_{0}$ due to thermal broadening of the mechanical linewidth^27^. We deduce ${g}_{0}/2\pi =84\,{\rm{kHz}}$ with ${C}_{0}$ value obtained from the efficiency measurement. After removing the independently measured ${\eta }_{{\rm{o}}}$ and ${\eta }_{{\rm{oc}}}$ from ${\eta }_{{\rm{e}}}$, we calculate ${\eta }_{{\rm{m}}}=9.9\times 1{0}^{-4} \sim 0.1 \%$ and the corresponding ${\gamma }_{\upmu }/2\pi$ = 1.9 kHz, about five orders of magnitude increase from our previous demonstration^27^. Improving the IDT-OMC mechanical frequency mismatch of $\Delta f=8.3\,{\rm{MHz}} \sim 2.5B$, would increase ${\eta }_{{\rm{m}}}$ by an order of magnitude. We note a $\sim \!{10} \%$ difference between ${\eta }_{{\rm{m}}}$ measured from acousto-optic modulation and ${\eta }_{{\rm{m}}}$ measured from conversion efficiencies. An overestimated ${g}_{0}$, as well as device aging between measurements affecting the frequency mismatch, explains this discrepancy in ${\gamma }_{\upmu }$.

In optical-to-microwave conversion, due to the much lower energy per photon at microwave frequency, a $\sim \!-50\,{\rm{dB}}$ reduction of the microwave signal power with respect to optical is incurred. With sub-microwatt optical sideband power, it becomes technically challenging to keep the converted microwave power above the VNA internal cross talk level. We adopt a more sensitive measurement scheme^22^ that uses a signal generator for the EOM input and an isolated real-time spectrum analyzer (RSA) for sensitive detection of the microwave output. The optical input sideband is calibrated by a fiber Fabry–Pérot tunable filter (FFP-TF) and is described in detail in Supplementary Note 9. The calibrated optical-to-microwave conversion efficiencies ${\eta }_{{\rm{eo}}}$ are plotted in Fig. 4a for comparison. Higher efficiencies are observed for blue-detuned ${\eta }_{{\rm{eo}}}$ than ${\eta }_{{\rm{oe}}}$. We attribute the difference to temperature fluctuation and device aging during the modification of measurement setup, which could lead to a different frequency mismatch between the OMC mechanical mode and a nearby waveguide mode.

To achieve bidirectional conversion measurement with the VNA, we fix the maximal pump laser power to $\sim$300 µW  limited by the setup and change the detuning $2\pi \times 1\,{\rm{GHz}}\,{<}\,| \Delta | \le {\omega }_{{\rm{m}}}$ to further increase the intracavity photon number ${n}_{{\rm{c}}}$. The measured conversion efficiencies are shown in Fig. 4b. Reasonable agreement between the two conversion directions is observed up to the phonon lasing regime, with highest stable conversion efficiency $\eta \approx 5.5 \%$ in the linear operation regime. The corresponding internal gain is ${G}_{{\rm{int}}}\approx 21\,{\rm{dB}}$. Figure 4c shows the measured $S$ parameters at the highest achieved efficiency. We obtain good agreement between optical-to-microwave and microwave-to-optical scattering parameters for both the lineshape and the peak efficiency. When ${n}_{{\rm{c}}}$ is further increased such that $C \, > \, 1$, we observe phonon lasing and amplification for both input optical sideband and input microwave signal. Operation in this nonlinear regime will be studied in future work.

## Discussion

To compare existing piezo-optomechanical transducers, we summarize parameters from various demonstrations in Table 1. ${\eta }_{{\rm{m}}}$ and ${P}_{{\rm{pump}}}$ from Vainsencher et al.^19^ are estimated from their measured total efficiency, intracavity photon number and other system parameters, assuming a grating coupler efficiency of $\sim \!{20} \%$. The ${\eta }_{{\rm{m}}}$ from Balram et al.^23^ is calculated from the measured driven coherent phonon population. We note that Shao et al.^28^ achieved an efficiency of $17 \%$ between microwave and mechanics, orders of magnitude higher than other approaches but at the cost of a reduced overlap between the optical and mechanical modes leading to a lower optomechanical coupling rate ${g}_{0}/2\pi$ = 1.1 kHz. They estimated a conversion efficiency ${\eta }_{{\rm{blue}}}=1.7\times 1{0}^{-5}$ from their system parameters. We define the on-chip single-pump-photon conversion efficiency as ${\eta }_{0}\equiv 4{\eta }_{{\rm{o}}}{\eta }_{{\rm{m}}}{C}_{0}$. The transducer reported here achieved roughly one order of magnitude higher ${\eta }_{0}$ comparing to all other demonstrations. The fiber-to-chip optical coupling efficiency ${\eta }_{{\rm{oc}}}$ is a separate issue and is not explicitly included in Table 1, though it affects the total efficiency numbers.

**Table 1:** Comparison of integrated piezo-optomechanical transducers.

Reference:	Vainsencher et al.^19^	Balram et al.^23,24^	Forsch et al.^25^	Jiang et al*.*^27^	Shao et al.^28^	This work
Platform:	AlN	GaAs	GaAs ($20$ mK)	LN	LN	LN
${g}_{0}/2\pi$ (Hz)	$1.1\,{\times} 1{0}^{5}$	$1.1\,{\times} 1{0}^{6}$	${\bf{1.3}}\,{\times} {{\bf{10}}}^{{\bf{6}}}$	$1.2\times 1{0}^{5}$	$1.1\,{\times} 1{0}^{3}$	$8\,{\times} 1{0}^{4}$
$\kappa /2\pi$ (Hz)	$1.5\,{\times} 1{0}^{10}$	$5.2\,{\times} 1{0}^{9}$	$5.8\times 1{0}^{9}$	$7.8\times 1{0}^{8}$	${\bf{9.5}}\,{\times} {{\bf{10}}}^{{\bf{7}}}$	$1.2\,{\times} 1{0}^{9}$
$\gamma /2\pi$ (Hz)	$5\,{\times} 1{0}^{6}$	$1.7\,{\times} 1{0}^{6}$	${\bf{2}}\,{\times} {{\bf{10}}}^{{\bf{5}}}$	$5\times 1{0}^{5}$	$1.3\,{\times} 1{0}^{6}$	$1.9\,{\times} 1{0}^{6}$
${C}_{0}\equiv 4{g}_{0}^{2}/(\kappa \gamma )$	$7\,{\times} 1{0}^{-7}$	$5.4\,{\times} 1{0}^{-4}$	${\bf{5.9}}\,{\times} {{\bf{10}}}^{-{\bf{3}}}$	$1.5\,{\times} 1{0}^{-4}$	$4\,{\times} 1{0}^{-8}$	$1.2\,{\times} 1{0}^{-5}$
${\eta }_{{\rm{o}}}\equiv {\kappa }_{{\rm{e}}}/\kappa$	${\bf{69}} \%$	$23 \%$	$65 \%$	$29 \%$	$15 \%$	$66 \%$
${\eta }_{{\rm{m}}}\equiv {\gamma }_{\upmu }/\gamma$	$\sim \!2\,{\times} 1{0}^{-4}$	$3\,{\times} 1{0}^{-10}$	$3.6\,{\times} 1{0}^{-10}$	$1.8\,{\times} 1{0}^{-8}$	${\bf{1.7}}\,{\times} {{\bf{10}}}^{-{\bf{1}}}$	$1{0}^{-3}$
${\eta }_{0}\equiv 4{\eta }_{{\rm{o}}}{\eta }_{{\rm{m}}}{C}_{0}$	$\sim \!3.7\,{\times} 1{0}^{-10}$	$1.5\,{\times} 1{0}^{-13}$	$5.5\,{\times} 1{0}^{-12}$	$3\times 1{0}^{-12}$	$4\,{\times} 1{0}^{-9}$	${\bf{3.2}}\,{\times} {{\bf{10}}}^{-{\bf{8}}}$
${\eta }_{{\rm{int}}}\equiv 4C/{(1+C)}^{2}$	$6.5\,{\times} 1{0}^{-3}$	–	${\bf{7.2}}\,{\times} {{\bf{10}}}^{-{\bf{2}}}$	$\sim \!1{0}^{-2}$	$7\,{\times} 1{0}^{-4}$	$2.6\,{\times} 1{0}^{-2}$
${\eta }_{{\rm{eo}}}$	$9\,{\times} 1{0}^{-8}$	–	–	–	–	${\bf{1.1}}\,{\times} {{\bf{10}}}^{-{\bf{5}}}$
${\eta }_{{\rm{oe}}}$	$2\,{\times} 1{0}^{-8}$	–	$5.5\,{\times} 1{0}^{-12}$	$\sim \!1{0}^{-10}$	–	${\bf{1.1}}\,{\times} {{\bf{10}}}^{-{\bf{5}}}$
${\eta }_{{\rm{blue}}}$	–	–	–	–	$1.7\,{\times} 1{0}^{-5}$	${\bf{5.5}}\,{\times} {{\bf{10}}}^{-{\bf{2}}}$
${P}_{{\rm{pump}}}$	$\sim$60 µW (${\eta }_{{\rm{eo}}}$) $\sim$ 110 µW (${\eta }_{{\rm{oe}}}$)	–	$\sim \!{\bf{0.5}}$ µW	$\sim$3 µW	1 mW	3.3 µW (red) 323 µW (blue)
${E}_{{\rm{bit}}}$ (J)	$1.4\,{\times} 1{0}^{-11}$	$7.5\,{\times} 1{0}^{-9}$	$6.3\,{\times} 1{0}^{-9}$	$2\,{\times} 1{0}^{-9}$	$1.4\,{\times} 1{0}^{-11}$	${\bf{9.7}}\,{\times} {{\bf{10}}}^{-{\bf{14}}}$
${E}_{{\rm{qubit}}}$ (J)	$1.3\,{\times} 1{0}^{-6}$	$8.3\,{\times} 1{0}^{-3}$	$9.7\,{\times} 1{0}^{-4}$	$1.9\,{\times} 1{0}^{-4}$	$7.8\,{\times} 1{0}^{-9}$	${\bf{3.5}}\,{\times} {{\bf{10}}}^{-{\bf{9}}}$

${\gamma }_{\upmu }$ is defined as the decay rate from the local mechanical mode to the 50 $\Omega$ microwave channel. Optical-to-microwave (${\eta }_{{\rm{eo}}}$), microwave-to-optical (${\eta }_{{\rm{oe}}}$) and blue-side-pump (${\eta }_{{\rm{blue}}}$) efficiencies are listed separately. We note that only the demonstrations on LN platform are in the sideband-resolved regime, and the corresponding ${E}_{{\rm{bit}}}$ is calculated differently (see the main text). The values most favorable for efficient conversion are highlighted in bold.

As a modulator for classical optical fields, our device exhibits both a ${V}_{\pi }$ and an energy-per-bit ${E}_{{\rm{bit}}}{\hskip -1pt}$^3,5^ that is orders of magnitude lower than previous acousto-optic demonstrations. We use Eqs. (2) and (3) to estimate the energy efficiency of a range of devices. In general, the piezo-optomechanical devices lag significantly behind the best electro-optic devices^41–44^. By further improving the electrical-to-mechanical efficiency from ${\eta }_{{\rm{m}}} \sim 1{0}^{-3}$ to closer to unity, we expect to push the classical performance of the device deep into the sub-femtojoule regime where performance in excess of the electro-optic systems becomes possible. Implementing a similar approach in a hybrid platform that integrates silicon with lithium niobate^45,46^ would allow even greater improvements by increasing ${g}_{0}$ by an order of magnitude and enabling sub-attojoule modulation energy. Finally, we stress that the low dissipated energies-per-bit are not directly related to the limited mechanical bandwidth, but rather mostly as a result of the strong optomechanical interaction. The driving bandwidth could be increased significantly by advances in OMC and IDT design for applications requiring faster modulation while keeping a low dissipated energy-per-bit.

For quantum transduction, each converted qubit comes at a cost of optical pump power dissipated in the fridge ${E}_{{\rm{qubit}}}=\hslash {\omega }_{{\rm{c}}}\kappa {\kappa }_{{\rm{i}}}/{g}_{0}^{2}/(4{\eta }_{{\rm{o}}}{\eta }_{{\rm{m}}})$, where ${\kappa }_{{\rm{i}}}$ is the intrinsic optical cavity loss rate (see refs. ^5,47^ and also Supplementary Note 6). Cooling our transducer to cryogenic temperature would reduce material loss for the IDT and the OMC, and may increase both ${\eta }_{{\rm{m}}}$ and ${C}_{0}$ by more than one order of magnitude. Adding another order of magnitude from matching IDT and OMC mechanical frequencies, we expect a picojoule energy-per-qubit and more than three orders of magnitude better efficiency to be possible, bringing the total efficiency with a red-detuned pump up to $\gtrsim 1 \%$ with only $\sim \!{500}$ intracavity photons and corresponding optical pump power ${P}_{{\rm{pump}}}\approx$ 3.3 µW. When the transducer is further resonantly coupled to a superconducting qubit/resonator with a characteristic impedance ${Z}_{{\rm{c}}} \sim \ 300\ \Omega$, we estimate the coupling rate between the qubit/resonator and the OMC mechanical mode to be ${g}_{\upmu }=\sqrt{{\gamma }_{\upmu }{\omega }_{{\rm{m}}}}\cdot \sqrt{{Z}_{{\rm{c}}}/{Z}_{0}}/2 \sim 2\pi \times$ 2.3 MHz^27^, putting us in the strong coupling regime so long as the qubit/resonator linewidth ${\kappa }_{\upmu }/2\pi$ < 2.3 MHz.

In conclusion, we designed and fabricated an integrated piezo-optomechanical transducer by combining an efficient wavelength-scale mechanical waveguide transducer and an optimized optomechanical crystal on LNOS platform. The microwave-to-mechanical conversion efficiency is increased by a factor of $1{0}^{5}$ comparing to our previous design without severely impacting the optomechanical coupling or dissipation. We demonstrated efficient acousto-optic modulation with ${V}_{\pi }=24\,{\rm{mV}}$, bidirectional conversion efficiency of $1{0}^{-5}$ with 3.3 µW  red-detuned optical pump and $5.5 \%$ with 323 µW  blue-detuned pump at room temperature. We expect our transducers to have reduced material loss and an increased efficiency at cryogenic temperature, opening up experiments in the quantum regime between optical photons, microwave photons and phonons, and superconducting qubits^30,31^.

## Supplementary information

Supplementary Information

Peer Review File

## Data Availability

All data that support the findings of this study are available in the main text, Supplementary Information, as well as from the corresponding authors upon reasonable request.
