# Supplementary material for: Efficient bidirectional piezo-optomechanical transduction between microwave and optical frequency
Source: Nat Commun. 2020 Mar 3;11:1166. doi: 10.1038/s41467-020-14863-3 (PMC7054291; doi:10.1038/s41467-020-14863-3)
Supplement: Supplementary file 1 — Supplementary Information [file 41467_2020_14863_MOESM1_ESM.pdf]

# Supplementary Information for Efficient bidirectional piezo-optomechanical transduction between microwave and optical frequency

Wentao Jiang,* Christopher J. Sarabalis, Yanni D. Dahmani, Rishi N. Patel, Felix M. Mayor, Timothy P. McKenna, Raphaël Van Laer, and Amir H. Safavi-Naeini†
*Department of Applied Physics and Ginzton Laboratory, Stanford University, 348 Via Pueblo Mall, Stanford, California 94305, USA*
(Dated: February 3, 2020)

## Supplementary Note 1. LINEARIZED OPTOMECHANICAL SYSTEM WITH BLUE-DETUNED OPTICAL PUMP

In this section we derive the semi-classical theory for the linear response of an optomechanical crystal pumped on the blue side. Starting with the Hamiltonian describing an optomechanical system

$$H = \hbar\omega_c a^\dagger a + \hbar\omega_m b^\dagger b + \hbar g_0 a^\dagger a(b + b^\dagger), \tag{1}$$

the corresponding Heisenberg-Langevin equations of motion is

$$\dot{a} = -(i\Delta + \kappa/2)a - ig_0 a(b + b^\dagger) - \sqrt{\kappa_e} a_{in}, \tag{2}$$
$$\dot{b} = -(i\omega_m + \gamma/2)b - ig_0 a^\dagger a - \sqrt{\gamma_e} b_{in}, \tag{3}$$

where we have included the loss and input of the optical and the mechanical mode, and moved to a rotating frame following the pump laser frequency.

Following the derivation in Ref. [1], we substitute the operators with relevant amplitudes,

$$a \to \alpha_0 e^{-i\omega_p t} + \alpha_- e^{-i(\omega_p+\omega)t} + \alpha_+ e^{-i(\omega_p-\omega)t}, \tag{4}$$
$$b \to \beta_0 + \beta_- e^{-i\omega t}, \tag{5}$$

where $\omega_p$ is the optical pump frequency and $\omega$ is the microwave drive frequency, either to the EOM or to the IDT. For weak sideband amplitudes $\alpha_\pm \ll \alpha_0$, $\alpha_0$ is given by

$$\alpha_0 = \frac{-\sqrt{\kappa_e}\alpha_{in,p}}{i\Delta + \kappa/2}, \tag{6}$$

where $\alpha_{in,p}$ is the input optical pump amplitude. The resulting equations of motion for the sidebands are

$$\pm i\omega\alpha_\pm = -(i\Delta + \kappa/2)\alpha_\pm - ig_0\alpha_0\beta_\pm - \sqrt{\kappa_e}\alpha_{in,\pm}, \tag{7}$$
$$-i\omega\beta_- = -(i\omega_m + \gamma/2)\beta_- - ig_0(\alpha_0^*\alpha_- + \alpha_0\alpha_+^*) - \sqrt{\gamma_e}\beta_{in,-}, \tag{8}$$
$$\beta_+ \equiv \beta_-^*. \tag{9}$$

When we pump the optical mode at the blue side with $\Delta \equiv \omega_c - \omega_p \sim -\omega_m$ and drive at frequency $\omega \sim \omega_m$, the up-converted sideband $\alpha_-$ is negligible for a sideband-resolved system. For microwave-to-optical (optical-to-microwave conversion) process, $\alpha_+$ and $\beta_-$ are solved as a function of input $\beta_{in,-}$ ($\alpha_{in,+}$). The converted output field is given by $\alpha_{out,+} = \sqrt{\kappa_e}\alpha_+$ ($\beta_{out,-} = \sqrt{\gamma_e}\beta_-$).

For optical sideband input and readout ($\beta_{in,-} = 0, \alpha_{out,+} = \alpha_{in,+} + \sqrt{\kappa_e}\alpha_+$), we derive

$$S_{oo}(\omega) \equiv \frac{\alpha_{out,+}}{\alpha_{in,+}} = \frac{-\kappa_e}{i(\Delta + \omega) + \frac{\kappa}{2} - \frac{G^2}{i(\omega-\omega_m)+\gamma/2}}, \tag{10}$$

where $G = g_0|\alpha_0| = g_0\sqrt{n_c}$ is the effective optomechanical coupling rate. $n_c \equiv |\alpha_0|^2$ is the intracavity pump photon number. $S_{oo}$ is directly measured by the VNA and normalized by a background taken with the pump laser far-detuned

* wentao@stanford.edu
† safavi@stanford.edu

from the optical mode to remove the response from electronic components. We extract the pump detuning $\Delta$, optical mode decay rates $\kappa$ and $\kappa_\mathrm{e}$ from $S_\mathrm{oo}$. One example of the measured $S_\mathrm{oo}$ is shown in Supplementary Note 3.

Similarly, the microwave-to-optical and optical-to-microwave conversion scattering parameters can be solved,

$$S_\mathrm{oe} \equiv \frac{\alpha_\mathrm{out,+}}{\beta_\mathrm{in,+}} = \frac{\sqrt{\kappa_\mathrm{e}\gamma_\mathrm{e}}ig_0\alpha_0}{[i(\Delta+\omega)+\frac{\kappa}{2}][i(\omega-\omega_\mathrm{m})+\frac{\gamma}{2}]-G^2}, \tag{11}$$

$$S_\mathrm{eo} \equiv \frac{\beta_\mathrm{out,+}}{\alpha_\mathrm{in,+}} = \frac{-\sqrt{\kappa_\mathrm{e}\gamma_\mathrm{e}}ig_0\alpha_0^*}{[i(\Delta+\omega)+\frac{\kappa}{2}][i(\omega-\omega_\mathrm{m})+\frac{\gamma}{2}]-G^2}. \tag{12}$$

When the laser is locked to detuning $\Delta = -\omega_\mathrm{m}$ and the input frequency is at $\omega = \omega_\mathrm{m}$, the conversion efficiencies are simplified to

$$\eta_\mathrm{oe} = \eta_\mathrm{eo} = |S_\mathrm{oe}|^2 = |S_\mathrm{eo}|^2 \tag{13}$$

$$= \frac{\gamma_\mathrm{e}}{\gamma}\frac{\kappa_\mathrm{e}}{\kappa}\frac{4C}{(1-C)^2}, \tag{14}$$

where $C = 4G^2/(\kappa\gamma)$ is the cooperativity. In contrast to the efficiency with red-side-pump $\eta = \frac{\gamma_\mathrm{e}}{\gamma}\frac{\kappa_\mathrm{e}}{\kappa}\frac{4C}{(1+C)^2}$, the blue-side-pump efficiency blows up at $C = 1$. Phonon lasing results when $C \geq 1$ is achieved and the linear theory no longer applies to the system.

In the low cooperativity limit where $C \ll 1$, the conversion efficiency is approximately linear with respect to cooperativity and thus linear with respect to $n_\mathrm{c}$,

$$\eta_\mathrm{oe} = \eta_\mathrm{eo} = 4C\frac{\gamma_\mathrm{e}}{\gamma}\frac{\kappa_\mathrm{e}}{\kappa} = 4\frac{4g_0^2\gamma_\mathrm{e}\kappa_\mathrm{e}}{\gamma^2\kappa^2}n_\mathrm{c}. \tag{15}$$

For microwave input and output, we ignore the detailed IDT response for simplicity and combine the IDT mismatch, IDT transduction efficiency and the coupling between the mechanical waveguide and the local mechanical mode to a single parameter $\gamma_\mu$, defined as the mechanical decay rate from the local mechanical mode to the microwave transmission line. In this case, $\beta_\mathrm{in,\pm}$ can be treated as microwave input and output amplitudes, and $\gamma_\mathrm{e}$ is replaced by $\gamma_\mu$. This approximation is valid in the low microwave-to-mechanical conversion efficiency regime. A full analysis of a coupled three-mode system shows that the peak conversion efficiency is given by [2–4]

$$\eta = \frac{\kappa_\mathrm{e}}{\kappa}\frac{\kappa_\mathrm{c,e}}{\kappa_\mathrm{c}}\frac{4C_\mathrm{ab}C_\mathrm{bc}}{(1\pm C_\mathrm{ab}+C_\mathrm{bc})^2}, \tag{16}$$

where $C_\mathrm{ab} \equiv C$ is the cooperativity between mode $a$ and $b$, and $C_\mathrm{bc} \equiv 4g_\mathrm{bc}^2/(\kappa_\mathrm{c}\gamma)$ is the cooperativity between OMC mechanical mode $b$ and IDT electromechanical mode $c$. $g_\mathrm{bc}$ is the coupling rate between mode $b$ and $c$. $\kappa_\mathrm{c}$ and $\kappa_\mathrm{c,e}$ are the total and external decay rate of mode $c$ respectively. For weak coupling between $b$ and $c$ where $C_\mathrm{bc} \ll 1$, the efficiency can be approximated by

$$\eta \approx \frac{\kappa_\mathrm{e}}{\kappa}\frac{4g_\mathrm{bc}^2\kappa_\mathrm{c,e}/\kappa_\mathrm{c}^2}{\gamma}\frac{4C_\mathrm{ab}}{(1\pm C_\mathrm{ab})^2}. \tag{17}$$

By defining $\gamma_\mu \equiv 4g_\mathrm{bc}^2\kappa_\mathrm{c,e}/\kappa_\mathrm{c}^2$, we obtain the two-mode conversion situation with an effective decay rate $\gamma_\mu$ from the mechanical mode to the microwave transmission line. For $\kappa_\mathrm{c,e}/\kappa_\mathrm{c} \sim 1$, the weak-coupling condition $C_\mathrm{bc} \ll 1$ translates to $\gamma_\mu/\gamma \ll 1$, which is valid for the transducer in this work.

In the above derivation, a frequency mismatch $\Delta_\mathrm{bc}$ between the OMC mechanical mode and the IDT electromechanical mode is not considered. A non-zero $\Delta_\mathrm{bc}$ modifies $\gamma_\mu$ as

$$\gamma_\mu(\Delta_\mathrm{bc}) = \frac{4g_\mathrm{bc}^2\kappa_\mathrm{c,e}}{4\Delta_\mathrm{bc}^2+\kappa_\mathrm{c}^2} = \frac{1}{1+4\Delta_\mathrm{bc}^2/\kappa_\mathrm{c}^2}\gamma_\mu(0). \tag{18}$$

## Supplementary Note 2. MODE DECOMPOSITION OF THE IDT AND THE OMC LEAKAGE IN THE MECHANICAL WAVEGUIDE

We showed the simulated IDT and OMC modes in the mechanical waveguide region in the main text. Here we further perform mode decomposition of the leakage mode to the guided modes of the mechanical waveguide [5]. The resulting fractional powers in different waveguide modes are listed in Table Supplementary Table 1. The OMC leakage motion is decomposed after the curved waveguide.

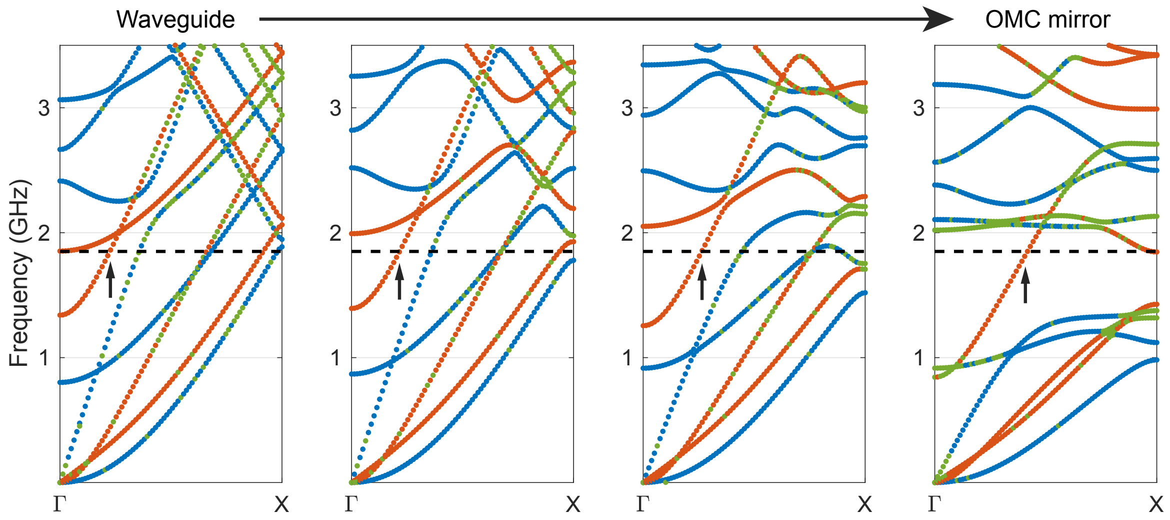


Supplementary Figure 1. Evolution of mechanical bands of the linear taper from the simple waveguide to the OMC mirror cell. Sub-figures from left to right correspond to band structures of the first taper unitcell with minor geometric change (left), the OMC mirror cell band structure (right) and two intermediate unitcells between the waveguide and the OMC mirror cell geometry. The bands are classified by semi-y-symmetric (blue), semi-y-asymmetric (red) and mixed (green). Horizontal dashed lines represent the OMC breathing mode frequency. Black arrows indicate the first-order longitudinal motion band.

TABLE Supplementary Table 1. Mode decomposition of the IDT and OMC leakage mode to the mechanical waveguide modes. Numbers in percentage are the fractional power in corresponding modes.

| Waveguide modes | OMC→WG | IDT→WG | IDT $\eta$ | $T_{\mathrm{b\mu}}$ |
|---|---|---|---|---|
| L1 | 64.1% | 74.6% | 0.6% | 0.19% |
| SH1 | 28.0% | $< 0.01\%$ | 0 | 0 |
| A2 | 5.4% | 4.37% | 0.04% | 0.013% |
| SH0 | 2.0% | $< 0.01\%$ | 0 | 0 |
| A1 | 0.1% | 17.9% | 0.14% | 0.044% |
| A0 | 0.2% | 0.4% | $< 0.01\%$ | 0 |
| L0 | $< 0.1\%$ | 2.7% | 0.02% | 0.006% |

We list seven most relevant waveguide modes in the table, including the first-order longitudinal (L1), first-order horizontal-shear (SH1), second-order Lamb (A2), fundamental horizontal-shear (SH0), first-order Lamb (A1), fundamental Lamb (A0) and fundamental longitudinal (L0). The higher order modes have one or more nodes along the in-plane transverse direction, because the waveguide width is much larger than the thickness.

Intuitively, the mechanical modes supported by the OMC mirror cell near the local breathing mode frequency are mostly asymmetric along the nanobeam symmetry plane. When the crystal mirror symmetry plane is aligned to the OMC mirror symmetry plane, the mirror cell modes near the OMC breathing mode frequency are strictly asymmetric [2]. As a result, the OMC mechanical leakage mostly enters the asymmetric waveguide modes such as L1 and SH1. The L1 mode has the smallest wave-vector, making it more strongly coupled to the Gamma-point OMC breathing mode. This is further illustrated in Supplementary Figure 1, where the evolution of the mechanical band structure from the waveguide geometry to the OMC mirror cell geometry is shown as four snapshots. There is no crystal symmetry along the geometric y-symmetry plane, but we could still classify the modes as semi-symmetric (blue), semi-asymmetric (red) or mixed (green) by evaluating proper overlap integrals. We observe that the L1 band always covers the OMC breathing mode frequency, indicated by the horizontal dashed lines and the black arrows.

In the IDT simulation, a material loss tangent that corresponds to a quality factor $Q_{\mathrm{i}} = 800$ is added to roughly match the simulated and measured peak conductance. We further define the IDT efficiency $\eta$ in terms of the fractional power in different waveguide modes over the total power dissipated in the IDT, including material loss and clamping loss. We list the simulated IDT $\eta$ in Table. Supplementary Table 1 and deduce that 99.2% of the total absorbed microwave power is dissipated in material loss and clamping. The material loss can be drastically eliminated by going to cryogenic temperature, while the clamping loss can be minimized by deploying phononic shield [6, 7]. In

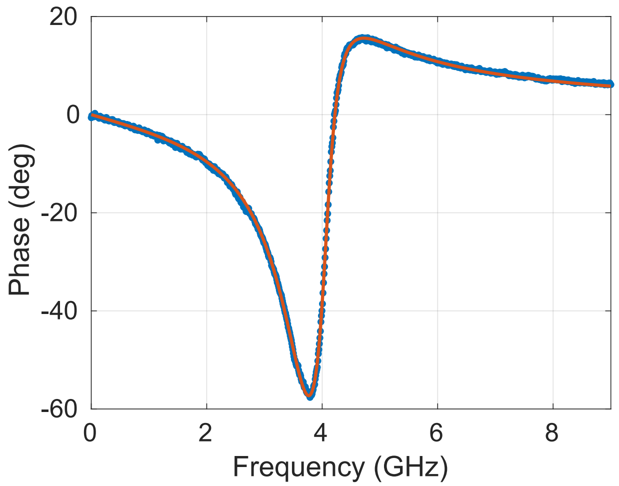


Supplementary Figure 2. Optical sideband response. Blue dots: measured phase response. Red curve: fit.

the actual device, 31.6% of the incident microwave power is absorbed by the transducer, from which we calculate the microwave-to-waveguide-mode scattering parameter $T_{b\mu}$. This is the ratio between the outgoing mechanical power in waveguide modes and the incident microwave power.

The measured microwave-to-mechanical conversion efficiency $\eta_{\mathrm{m}} \equiv \gamma_{\mu}/\gamma$ could be higher than the maximal $T_{b\mu}$ in the table. The OMC mechanical mode is coupled to multiple waveguide modes, and the standing wave resonances of the mechanical waveguide modify the overall response of the IDT-waveguide-OMC system.

## Supplementary Note 3. EXTRACTING PUMP DETUNING AND OPTICAL CAVITY DECAY RATES

We use the optical sideband response (optical to optical scattering parameter) of the device to extract the cavity-laser detuning $\Delta$ and the optical cavity decay rates $\kappa$ and $\kappa_{\mathrm{e}}$. Figure Supplementary Figure 2 shows a typical phase response of the optical-to-optical $S$ parameter. A fit curve based on the linearized optomechanical response theory is also shown as the red curve. The fitting method is discussed in detail in Ref. [8].

For this specific measurement, we extract a detuning $\Delta/2\pi = -3.698$ GHz, $\kappa/2\pi = 1203$ MHz and $\kappa_{\mathrm{e}}/2\pi = 781$ MHz. A significant phase response is the signature of an over-coupled optical mode where $\kappa_{\mathrm{e}} > \kappa/2$. A clear deviation from the measurement is observed if the fit curve is forced to have $\kappa_{\mathrm{e}} < \kappa/2$. The VNA parameters used are (power, bandwidth, average, resolution) = (0 dBm, 10 kHz, 10, 9 MHz).

## Supplementary Note 4. OPTOMECHANICAL BACKACTION MEASUREMENT

Here we show the optomechanical backaction measurement of the optomechanical coupling $g_0$ and the mechanical linewidth $\gamma$.

Supplementary Figure 3 shows the extracted mechanical linewidth versus intracavity photon number with blue detuned pump laser at $\Delta = -\omega_{\mathrm{m}}$. Blue curve is a linear fit, giving an intrinsic mechanical linewidth of $\gamma/2\pi = 1.93$ MHz and an optomechanical coupling rate $g_0/2\pi = 70$ kHz.

## Supplementary Note 5. EXTRACTING THE ACOUSTO-OPTIC MODULATION INDEX AND $V_{\pi}$

In this section we derive the optical cavity spectrum under acousto-optic modulation and show typical fit results for extracting the acousto-optic modulation index $h$.

When the optical cavity's resonant frequency $\omega_{\mathrm{c}}$ is being modulated, in the frame where the cavity frequency is fixed, the laser input is phase-modulated by the same strength and splits into sidebands with relative amplitudes given by Bessel functions of the first kind. We expect the output spectrum to be a convolution between the phase modulation combs and the Lorentzian cavity response. We derive the rigorous output spectrum in the following.

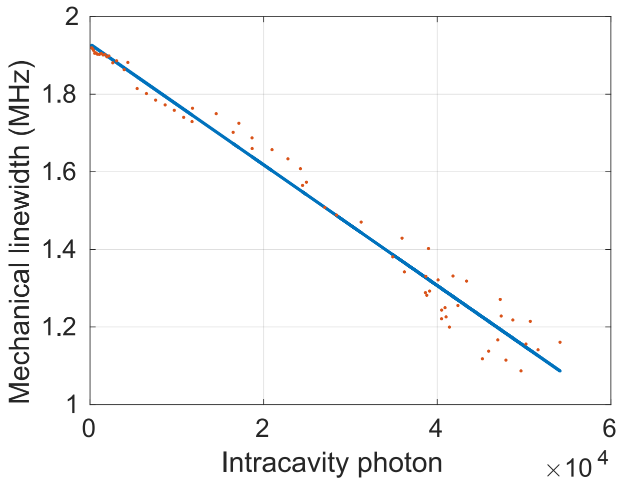


Supplementary Figure 3. Optomechanical backaction measurements and fit.

We start with the classical equation of motion for the optical field amplitude in the rotating frame of the laser,

$$\dot{\alpha} = -(i\Delta + \frac{\kappa}{2})\alpha - 2ig_0\sqrt{n_{\mathrm{phon}}}\cos(\omega_\mu t)\alpha - \sqrt{\kappa_\mathrm{e}}\alpha_{\mathrm{in}}, \tag{19}$$

where $g_0$ is the zero-point optomechanical coupling rate and $n_{\mathrm{phon}}$ is the intracavity phonon number from the microwave drive at frequency $\omega_\mu$. We use the following substitution to eliminate the time-dependent term on the right hand side:

$$\alpha \to \alpha' \exp[-ih\sin(\omega_\mu t)]. \tag{20}$$

We have introduced the acousto-optic modulation index $h \equiv 2g_0\sqrt{n_{\mathrm{phon}}}/\omega_\mu$. After the substitution, the equation for $\alpha'$ is

$$\dot{\alpha}' = -(i\Delta + \frac{\kappa}{2})\alpha' - \sqrt{\kappa_\mathrm{e}}\alpha_{\mathrm{in}}\exp(ih\sin\omega_\mu t) \tag{21}$$

$$= -(i\Delta + \frac{\kappa}{2})\alpha' - \sqrt{\kappa_\mathrm{e}}\alpha_{\mathrm{in}}\sum_n J_n(h)e^{in\omega_\mu t}, \tag{22}$$

where $J_n(h)$ are Bessel functions of the first kind. By separating $\alpha'$ further into sidebands $\alpha' = \sum_n \alpha'_n \exp(in\omega_\mu t)$, $\alpha'_n$ is time-independent and is given by

$$\alpha'_n = \frac{-\sqrt{\kappa_\mathrm{e}}\alpha_{\mathrm{in}}J_n(h)}{i(\Delta + n\omega_\mu) + \kappa/2}. \tag{23}$$

The output optical field is

$$\alpha_{\mathrm{out}} = \alpha_{\mathrm{in}} + \sqrt{\kappa_\mathrm{e}}\exp(-ih\sin\omega_\mu t)\sum_n \alpha'_n e^{in\omega_\mu t} \tag{24}$$

$$= \alpha_{\mathrm{in}}\exp(-ih\sin\omega_\mu t)\cdot \left(\sum_n J_n(h)e^{in\omega_\mu t} - \kappa_\mathrm{e}\sum_n \frac{J_n(h)e^{in\omega_\mu t}}{i(\Delta + n\omega_\mu) + \kappa/2}\right). \tag{25}$$

The slow photodetector used for the measurement selects the direct-current component of $|\alpha_{\mathrm{out}}|^2$. As a result, the

normalized reflection is a weighted sum of Lorentzians

$$\begin{aligned} R &\equiv \left\langle \left| \frac{\alpha_{\rm out}}{\alpha_{\rm in}} \right|^2 \right\rangle \\ &= \left\langle \left| \sum_n J_n(h) e^{in\omega_\mu t} \left( 1 - \frac{\kappa_{\rm e}}{i(\Delta + n\omega_\mu) + \kappa/2} \right) \right|^2 \right\rangle \\ &= \sum_n J_n(h)^2 \left| 1 - \frac{\kappa_{\rm e}}{i(\Delta + n\omega_\mu) + \kappa/2} \right|^2 \end{aligned} \tag{26}$$

We use Supplementary Equation 26 to fit the measured reflection spectrum and extract the acousto-optic modulation index $h$ for different microwave drive powers and frequencies as shown in the main text. We observed acousto-optic sidebands up to $h \sim 9$, hence the sum for $n$ is truncated at $\pm 20$, far enough comparing to the optical linewidth. The only fit parameters are $h$ and a frequency shift and scaling for compensating the difference between the laser wavelength readout and the microwave frequency. Scaling the frequency axis does not affect the unitless parameter $h$.

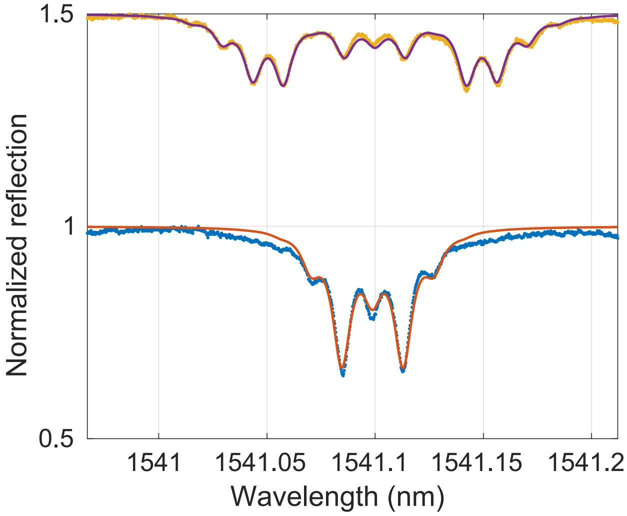


Supplementary Figure 4. Normalized optical reflection spectrum with acousto-optic modulation and corresponding fit curves.

Supplementary Figure 4 shows two typical reflection measurements and corresponding fit curves with $h = 1.747$ and $h = 4.812$ (shifted up by 0.5) respectively. Good fitting results are observed up to $h \sim 8$. For $h > 8$, the spectrum spreads out for a wide range of frequency, resulting in smaller reflection dips that are hard to be captured by the fit without a good initial guess.

The measured acousto-optic modulation index $h$ gives us a relation between the OM coupling $g_0$ and the microwave-mechanical external coupling rate $\gamma_\mu$. From standard input-output theory, the intracavity phonon number from input microwave photon flux $\dot{N}_{\rm in,\mu} = P_\mu/(\hbar\omega_\mu)$ is

$$n_{\rm phon} = \frac{\gamma_\mu \dot{N}_{\rm in,\mu}}{(\omega_{\rm m} - \omega_\mu)^2 + (\gamma/2)^2}. \tag{27}$$

When $\omega_\mu = \omega_{\rm m}$, the above expression is simplified to $n_{\rm phon} = 4\gamma_\mu \dot{N}_{\rm in,\mu}/\gamma^2$. Combining with the definition of modulation index $h$, we have

$$\gamma_\mu = \frac{h^2 \omega_\mu^2 \gamma^2}{16 g_0^2 \dot{N}_{\rm in,\mu}}. \tag{28}$$

Underestimation of $g_0$ and overestimation of $h$ and $\gamma$ could lead to a larger $\gamma_\mu$.

Since the extra phase variation of the cavity is given by $\phi(t) = h\sin(\omega_\mu t)$, it is natural to define the voltage required to obtain a $\pi$ phase shift as when $h_{\pi,\mathrm{PM}} \equiv 2g_0\sqrt{n_\mathrm{phon}}/\omega_\mu = \pi$ is achieved. The microwave voltage is implicitly included via the microwave power $P_\mu$ in $n_\mathrm{phon}$. Such a voltage is given by

$$V_{\pi,\mathrm{PM}} = \frac{h_{\pi,\mathrm{PM}}\sqrt{2P_\mu Z_0}}{h}, \tag{29}$$

where $P_\mu$ is the microwave power used to achieve the measured $h$. $Z_0 = 50\,\Omega$ is the impedance of the microwave transmission line.

We measured a maximal $h = 3.518$ with $P_\mu = 7.24\,\mu\mathrm{W}$. The deduced phase-modulation $V_{\pi,\mathrm{PM}}$ is 24.0 mV.

## Supplementary Note 6. ENERGY CONSUMPTION FOR CONVERTING ONE CLASSICAL BIT AND ONE QUANTUM BIT

### Encoding of classical information and relevant energy consumption

Consider encoding one bit of information onto an optical field via the optomechanical interaction where the frequency of the optical cavity is shifted by $g_0$ with the zero-point motion $x_\mathrm{zp}$ of the mechanical mode. When the mechanical motion is much slower than the dynamics of the optical cavity ($\omega_\mu \ll \kappa$), the optomechanical modulation of the cavity frequency can be approximated as quasi-static. When a coherent state is injected and reflected from the optical cavity, a displacement that is approximately $x_\pi = x_\mathrm{zp}(\kappa/2)/g_0$ gives a significant phase shift [9]. The corresponding mechanical energy is

$$E_\mathrm{mech} = \frac{1}{2}m_\mathrm{eff}\omega_\mathrm{m}^2 x_\pi^2 = \frac{1}{2}m_\mathrm{eff}\omega_\mathrm{m}^2 x_\mathrm{zp}^2\frac{\kappa^2}{4g_0^2} = \frac{\hbar\omega_\mathrm{m}\kappa^2}{16g_0^2}. \tag{30}$$

For an imperfect conversion between microwave and mechanical energy, the energy-per-bit is given by

$$E_\mathrm{bit} = \frac{E_\mathrm{mech}}{\eta_\mathrm{m}} = \hbar\omega_\mathrm{m}\frac{\kappa^2}{16g_0^2}\frac{1}{\eta_\mathrm{m}}. \tag{31}$$

In the sideband resolved regime where $\omega_\mathrm{m} \gtrsim \kappa$, the dynamics of the optical cavity has to be considered. For a coherent state $\alpha_0$ that is initially in the cavity, the classical equation of motion is

$$\dot{\alpha} = -\left(\frac{\kappa}{2} + 2ig_0\sqrt{n_\mathrm{phon}}\cos(\omega_\mathrm{m}t + \phi)\right)\alpha, \tag{32}$$

where we have chosen the frame rotating at the static cavity frequency $\omega_\mathrm{c}$. The information can be encoded in the phase factor with $\phi = 0$ or $\phi = \pi$. We choose this encoding method because it returns to a simple phase shift in the optical field in the quasi-static limit $\omega_\mathrm{m} \ll \kappa$. The time-dependent solution of the equation of motion is

$$\alpha(t) = \alpha_0\exp\left(-\frac{\kappa t}{2} - ih\sin(\omega_\mathrm{m}t + \phi)\right). \tag{33}$$

We have defined the modulation index $h \equiv 2g_0\sqrt{n_\mathrm{phon}}/\omega_\mathrm{m}$. The output field is given by $\alpha_\mathrm{out}(t) = \sqrt{\kappa}\alpha(t)$. We set $\kappa_\mathrm{e} = \kappa$ for simplicity.

In a completely classical theory of electromagnetics, an arbitrarily small change in the optical field can be in principle detected deterministically with a sufficiently sensitive measurement. Quantum noise limits imprecision of estimating observables such as the phase of the an optical field. For example the imprecision in phase of coherent states is given by the standard quantum limit (SQL) $\Delta\phi \approx 1/\sqrt{n}$, where $n$ is the average number of photons in the field [10]. Therefore, to determine whether a device has sufficiently changed the state of the field to encode a bit, we need to consider how an initial coherent state $|\alpha\rangle$ with fixed optical energy (here we take $n = |\alpha_0|^2 = 1$) is modified by the device and use the quantum detection theory [11] to calculate the probability of error $P_e$ in distinguishing resulting states $|\Psi_k\rangle$. Note that this error rate is absolutely the lowest that can be achieved given with any possible receiver. Since our optomechanical device is an open quantum system [12] where the field leaks out into a waveguide, we consider the output states to be in the Hilbert state of the all of the waveguide states, which is generated by an operator that is a superposition of the time-dependent output operators with a time-domain waveform that corresponds to the classical solution,

$$\hat{A} \equiv \int_0^\infty dt f^*(t)\hat{a}_\mathrm{out}(t). \tag{34}$$

Here $f(t) = \sqrt{\kappa}\exp\left(-\kappa t/2 - ih\sin(\omega_\mathrm{m}t+\phi)\right)$, and $\hat{a}_\mathrm{out}(t)$ is the annihilation operator for each time bin that obeys $[\hat{a}_\mathrm{out}(t), \hat{a}_\mathrm{out}(t')] = 0$ and $[\hat{a}_\mathrm{out}(t), \hat{a}^\dagger_\mathrm{out}(t')] = \delta(t-t')$. It is straightforward to verify that $\hat{A}$ is a properly normalized bosonic operator such that $[\hat{A}, \hat{A}] = 0$ and $[\hat{A}, \hat{A}^\dagger] = 1$. Note that the information is encoded in $\hat{A}$ via the phase factor $\phi$ in $f(t)$. From now on we explicitly denote the resulting operator with suffix as $\hat{A}_\phi$, where $\phi = 0$ or $\phi = \pi$. The commutation relations between operators with different values of $\phi$ are now $[\hat{A}_\phi, \hat{A}_{\phi'}] = 0$ and

$$[\hat{A}_\phi, \hat{A}^\dagger_{\phi'}] = \int_0^\infty f^*_\phi(t) f_{\phi'}(t) dt = \int_0^\infty dt \kappa e^{-\kappa t} e^{ih[\sin(\omega_\mathrm{m}t+\phi) - \sin(\omega_\mathrm{m}t+\phi')]}. \tag{35}$$

For an initial coherent state $|\alpha_0\rangle$ in the cavity, the output state is given by the coherent state generated by the operator $A_\phi$,

$$|\Psi_\phi\rangle = e^{-|\alpha_0|^2/2} e^{\alpha_0 \hat{A}^\dagger_\phi} |0\rangle . \tag{36}$$

We use $|0\rangle$ to represent the vacuum state in the continuous time domain.

A criterion is required to determine whether a bit of information is successfully encoded or not. It is naturally related to how separate are the two output state $|\Psi_0\rangle$ and $|\Psi_\pi\rangle$, and how well we could distinguish them by measurement which can be characterized by an error possibility $P_\mathrm{e}$ of misidentifying the state. The error possibility is bounded below by the Helstrom-Holevo lower bound [12]. For distinguishing two pure states with equal prior probability, the Helstrom-Holevo lower bound is given by

$$P_\mathrm{e} = \frac{1}{2}\left(1 - \sqrt{1-F}\right), \tag{37}$$

where $F \equiv |\langle\Psi_0|\Psi_\pi\rangle|^2$ is the fidelity between the two encoded states,

$$\begin{aligned} F &= \left| e^{-|\alpha_0|^2} \langle 0| e^{\alpha_0^* \hat{A}_0} e^{\alpha_0 \hat{A}_\pi} |0\rangle \right|^2 \\ &= \left| \exp\left[-|\alpha_0|^2 (1 - [\hat{A}_0, \hat{A}^\dagger_\pi])\right] \right|^2 . \end{aligned} \tag{38}$$

Intuitively, $P_\mathrm{e} = 0$ for two orthogonal state with $F = 0$ and $P_\mathrm{e} = 1/2$ for two identical state with $F = 1$. A low fidelity between the two states is desired for them to be more separable. For $F \ll 1$, $P_\mathrm{e} \approx F/4$. In addition, when a larger coherent state $\alpha_0$ is used, the more separate the two states are for fixed $n_\mathrm{phon}$ and $h$. It is important to consider the optical field used for the encoding, since a stronger field makes it easier to measure a smaller change in its properties, reducing the energy required to modify the optical material that is used for imposing the change.

### *Slow limit: $\omega_m \ll \kappa$*

Before we proceed with numerical calculation, it is instructive to look at the quasi-static or the slow-limit where $\omega_\mathrm{m} \ll \kappa$. In this situation, the difference between operators $\hat{A}_0$ and $\hat{A}_\pi$ is approximately a phase shift $\Delta\phi = \pm ih\omega_\mathrm{m}t$. The commutator and the fidelity are simplified to

$$[\hat{A}_0, \hat{A}^\dagger_\pi] = \int_0^\infty dt \kappa e^{-\kappa t} e^{2ih\sin\omega_\mathrm{m}t} \approx \frac{\kappa}{\kappa - 2ih\omega_\mathrm{m}}, \tag{39}$$

$$F \approx \exp\left(-2|\alpha_0|^2 \frac{h^2\omega_\mathrm{m}^2}{h^2\omega_\mathrm{m}^2 + (\kappa/2)^2}\right) = \exp\left(-2|\alpha_0|^2 \frac{4g_0^2 n_\mathrm{phon}}{4g_0^2 n_\mathrm{phon} + (\kappa/2)^2}\right). \tag{40}$$

As a result, the fidelity is limited by $F_\mathrm{min} = \exp(-2|\alpha_0|^2)$ and the characteristic modulation index for achieving a low fidelity and a low error probability occurs at $2g_0\sqrt{n_\mathrm{phon}} = \kappa/2$. The corresponding $F \approx \exp(-|\alpha_0|^2)$, $n_\mathrm{phon} = \kappa^2/(16g_0^2)$ and

$$E_\mathrm{bit,slow} = \frac{\hbar\omega_\mathrm{m} n_\mathrm{phon}}{\eta_\mathrm{m}} = \hbar\omega_\mathrm{m} \frac{\kappa^2}{16g_0^2}\frac{1}{\eta_\mathrm{m}}, \tag{41}$$

which is identical to Supplementary Equation 31. For an initial coherent state with an average photon number $|\alpha_0|^2 = 1$, we calculate the fidelity $F = 1/e$ and the error probability $P_\mathrm{e} = 10.25\%$.

*Fast limit: $\omega_{\rm m} \gg \kappa$*

Now we consider the fast-limit where $\omega_{\rm m} \gg \kappa$. By expanding the sinusoidal phase modulation into sidebands, the commutator and the fidelity are approximated by

$$[\hat{A}_0, \hat{A}_\pi^\dagger] = \int_0^\infty dt \kappa e^{-\kappa t} \sum_n J_n(2h) e^{in\omega_{\rm m} t} = \sum_n J_n(2h) \frac{\kappa}{\kappa - in\omega_{\rm m}} \approx J_0(2h), \tag{42}$$

$$F \approx \exp\left(-2|\alpha_0|^2 (1 - J_0(2h))\right). \tag{43}$$

$J_n$ is the Bessel function of the first kind. The minimal fidelity and minimal error probability are achieved at the first minimal of $J_0(2h) \approx -0.4$, where $2h \approx 3.832$. Comparing to the slow-limit situation, the fast-limit fidelity is much lower at this optimal modulation index. To have a better comparison among the slow-limit, fast-limit and the general situation, we look for the modulation index where $J_0(2h) \sim 1/2$ instead of the optimal modulation index, which gives a fidelity $F \approx \exp(-|\alpha_0|^2)$, similar to the slow-limit fidelity. The corresponding $h \approx 0.76$, $n_{\rm phon} = h^2\omega_{\rm m}^2/(4g_0^2) \approx \omega_{\rm m}^2/(8g_0^2)$ and

$$E_{\rm bit,fast} = \frac{\hbar\omega_{\rm m} n_{\rm phon}}{\eta_{\rm m}} = \hbar\omega_{\rm m} \frac{\omega_{\rm m}^2}{8g_0^2} \frac{1}{\eta_{\rm m}}. \tag{44}$$

*Numerical calculation for the general situation*

In the general case, we assume that the best measurement is adopted to achieve the Helstrom-Holevo lower bound and we look at the modulation index $h$ or equivalently the intracavity phonon number $n_{\rm phon}$ that is required to reach a given error rate. We evaluate Supplementary Equation 38 and Supplementary Equation 35 numerically for different ratio between $\omega_{\rm m}$ and $\kappa$, and we search for the $n_{\rm phon}$ required to reach $P_{\rm e} = 10\%$ for an initial coherent state with an average photon number $|\alpha_0|^2 = 1$.

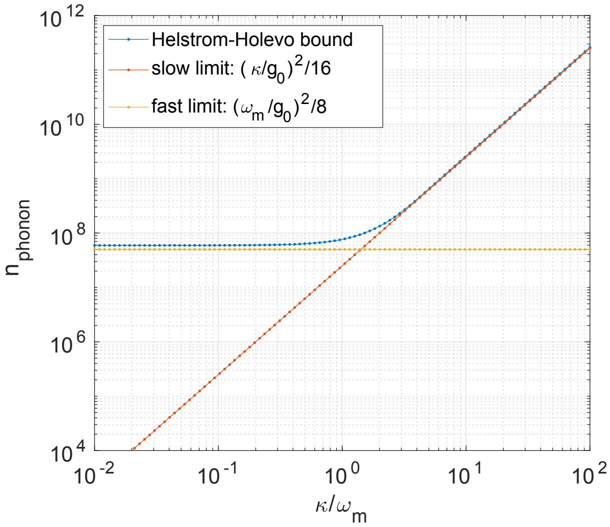


Supplementary Figure 5. Number of intracavity phonons required to achieve an error probability $P_{\rm e} = 10\%$ for distinguishing between two encoded states with an initial coherent state which has an average photon number $|\alpha_0|^2 = 1$ in the optical cavity. Blue line is the numerical calculation from Helstrom-Holevo bound. Red (yellow) line is the approximated result in the slow (fast) limit where $\omega_{\rm m} \ll \kappa$ ($\omega_{\rm m} \gg \kappa$).

We show the calculated $n_{\rm phon}$ in Supplementary Figure 5 for fixed $g_0$ and $\omega_{\rm m}$, and varying $\kappa$ by four orders of magnitude. We adopt $g_0/\omega_{\rm m} = 5 \times 10^{-5}$ for the calculation, which closely represents the device demonstrated in this work. We clearly observe that $n_{\rm phon}$ given by the Helstrom-Holevo bound is well approximated by the slow and fast limit in the corresponding regimes. Interestingly, for a sideband-unresolved piezo-optomechanical transducer device with a given mechanical mode frequency $\omega_{\rm m}$ and an optomechanical coupling $g_0$, decreasing the optical linewidth lowers the energy per bit until $\kappa \sim \omega_{\rm m}$. Further increasing the optical quality factor no longer helps for reducing the energy consumption.

**Energy consumption in quantum frequency conversion**

In the case of quantum transduction, an unity internal conversion efficiency is desired. For low microwave-to-mechanical conversion efficiency, best internal conversion is achieved at optomechanical cooperativity $C \equiv 4g_0^2 n_c/(\kappa\gamma) = 1$. The non-zero optical cavity internal decay rate $\kappa_i$ leads to a power dissipation

$$P_{\mathrm{diss}} = \hbar\omega_c n_c \kappa_i. \tag{45}$$

The conversion rate is limited by the mechanical-to-microwave decay rate $\gamma_\mu$, leading to the energy-per-qubit

$$\frac{P_{\mathrm{diss}}}{\gamma_\mu} = \frac{\hbar\omega_c \kappa\kappa_i}{4g_0^2\eta_m}. \tag{46}$$

However, the converted photon only successfully leaves the converter at efficiency $\eta_o \equiv \kappa_e/\kappa$, adding another factor to the actual energy-per-qubit,

$$E_{\mathrm{qubit}} = \hbar\omega_c \frac{\kappa\kappa_i}{4g_0^2}\frac{1}{\eta_m\eta_o}. \tag{47}$$

## Supplementary Note 7. MICROWAVE-TO-OPTICAL CONVERSION EFFICIENCY CALIBRATION

The microwave-to-optical conversion efficiency is defined as

$$\eta_{\mathrm{oe}} \equiv \frac{\dot{N}_{\mathrm{out,o}}}{\dot{N}_{\mathrm{in,\mu}}}, \tag{48}$$

where $\dot{N}_{\mathrm{out,o}}$ is the output optical sideband photon flux after the photons enter the lensed fiber, and $\dot{N}_{\mathrm{in,\mu}}$ is the input microwave photon flux before the IDT. $\eta_{\mathrm{oe}}$ is the total efficiency including the IDT mismatch and transduction efficiency, and the fiber-to-chip optical coupling efficiency $\eta_{\mathrm{oc}} \sim 65\%$.

$\dot{N}_{\mathrm{in,\mu}}$ is given by

$$\dot{N}_{\mathrm{in,\mu}} = \eta_{\mathrm{cable}} P_{\mathrm{in,\mu}}/(\hbar\omega_\mu), \tag{49}$$

where $\eta_{\mathrm{cable}} = 57.5\%$ is the microwave cable loss and $P_{\mathrm{in,\mu}} = -50$ dBm is the VNA output power.

$\dot{N}_{\mathrm{out,o}}$ is calibrated using the method introduced in Ref. [13] and also briefly described here. After each measurement, the pump laser is detuned from the optical cavity, and a second laser with optical power $P_{\mathrm{cal,o}} \sim 150$ nW $\ll P_{\mathrm{in}}$ is tuned to $\sim \omega_m$ away from the pump laser without changing any other settings in the measurement setup. The beat tone between the pump laser and the calibration laser is measured by the highspeed detector and the RSA. The pump laser power $P_{\mathrm{in}}$ is then fully attenuated to $\ll 1$ nW, and the calibration laser power $P_{\mathrm{cal,o}}$ is measured by a sensitive power meter (PM).

By integrating the microwave power of the beat tone $P_{\mathrm{cal,\mu}}$ on the RSA and measure the optical insertion loss $\eta_{\mathrm{out}} = 63.6\%$ from the lensed fiber to the power meter, we get the optical detection gain

$$G \equiv \frac{P_{\mathrm{cal,\mu}}}{P_{\mathrm{cal,o}}/\eta_{\mathrm{out}}}, \tag{50}$$

which is between the optical power at the lensed fiber output and the microwave power at the RSA.

We measure the converted optical sideband using the highspeed detector and the RSA with VNA output frequency fixed at the peak conversion frequency. The measured microwave power $P_{\mathrm{out,\mu}}$ on RSA is then converted to optical sideband power $P_{\mathrm{out,o}}$ at the lens fiber output using the calibrated detection gain $G$. The converted sideband photon flux is then calculated by

$$\dot{N}_{\mathrm{out,o}} = \frac{P_{\mathrm{out,o}}}{\hbar\omega_c} = \frac{P_{\mathrm{out,\mu}}}{G\hbar\omega_c}. \tag{51}$$

## Supplementary Note 8. OPTICAL-TO-MICROWAVE CONVERSION EFFICIENCY CALIBRATION

The optical-to-microwave conversion efficiency is defined similar to Supplementary Equation 48 as

$$\eta_{\mathrm{eo}} \equiv \frac{\dot{N}_{\mathrm{out},\mu}}{\dot{N}_{\mathrm{in,o}}}. \tag{52}$$

$\dot{N}_{\mathrm{in,o}}$ is the input optical sideband photon flux before the lensed fiber and $\dot{N}_{\mathrm{out},\mu}$ is the converted microwave photon flux at the IDT output.

Given the measured $S_{21}$ parameter on the VNA and the VNA output power to EOM $P_{\mathrm{EOM},\mu}$, we calculate the output microwave flux from the device

$$\dot{N}_{\mathrm{out},\mu} = \frac{P_{\mathrm{EOM},\mu}|S_{21}|^2}{\eta_{\mathrm{cable}}\hbar\omega_{\mu}}. \tag{53}$$

To calibrate the input optical sideband flux, we use a 1% beamsplitter at the EOM output and a fiber Fabry-Pérot tunable filter (FFP-TF, Micron Optics), and scan the FFP-TF to pickup the pump and sidebands separately. The filter output is detected by a Newport Nanosecond Photodetector, amplified and recorded.

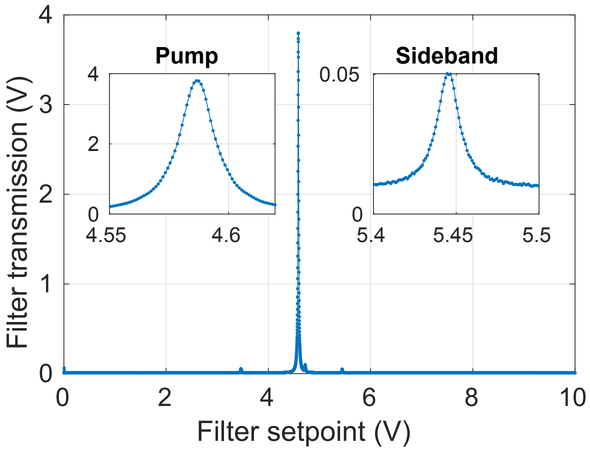


Supplementary Figure 6. A typical filter scan. Left inset: zoomed-in plot of the pump. Right inset: zoomed-in plot of the lower frequency sideband.

A typical filter scan result is shown in Supplementary Figure 6. Higher filter setpoint voltage corresponds to longer filter pass wavelength. Two sidebands generated by the electro-optic amplitude modulation are clearly visible with good signal-to-noise ratio. The small peak near the pump corresponds to a filter Fabry-Pérot cavity mode with a different polarization and is nearly fully suppressed. The sideband ratio $r \equiv P_{\mathrm{sb}}/P_{\mathrm{pump}}$ is measured from the ratio between the peak voltages of the relevant sideband and the pump, respectively. The dark voltage is subtracted before taking the ratio. Using 8 dBm VNA output microwave power to the EOM, we measure $r = (1.41 \pm 0.08)\%$. The other VNA parameters are (bandwidth, average, resolution) = (100 Hz, 4, 10 kHz). The filter scan is taken for every different optical-to-microwave conversion measurement. Combining the sideband ratio and the measured input pump power $P_{\mathrm{in,p}}$ before the lensed fiber, the input sideband photon flux is

$$\dot{N}_{\mathrm{in,o}} = \frac{P_{\mathrm{in,sb}}}{\hbar\omega_{\mathrm{c}}} = \frac{P_{\mathrm{in,p}}r}{\hbar\omega_{\mathrm{c}}}. \tag{54}$$

## Supplementary Note 9. OPTICAL-TO-MICROWAVE CONVERSION MEASUREMENT WITH LOW OPTICAL PUMP POWER

Due to a much lower energy per photon at the microwave frequency, the optical-to-microwave conversion is difficult to measure with VNA at low optical pump power. We adopt a different setup, using a microwave signal generator (SG) for the optical sideband generation and the RSA for detection of the converted microwave signal (Supplementary

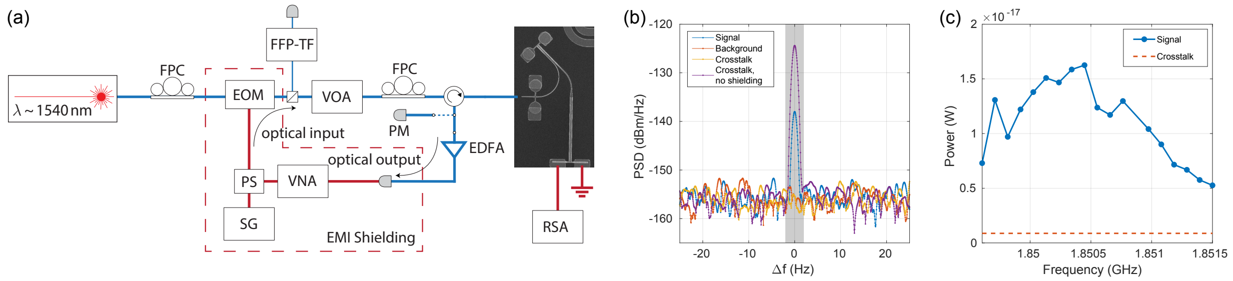


Supplementary Figure 7. Low pump power optical-to-microwave conversion measurement. (a) Measurement setup. (b) A typical power spectral density (PSD) of the converted microwave signal. The RSA background and the crosstalk are also shown for comparison. The crosstalk is measured with SG on and zero input optical power. The EMI shielding reduced the crosstalk by more than 30 dB. The shaded region shows the frequency range used for integrating the total microwave power. (c) Converted microwave power versus microwave frequency, from which a peak conversion efficiency $\eta_{\mathrm{eo}} = 1.09\times10^{-5}$ is extracted.

Figure 7(a)). The maximal output power of the SG is $P_{\mathrm{max}} = 25$ dBm and is much higher than the maximal output power of the VNA, bringing the optical sideband ratio $r$, defined as the ratio between the sideband optical power and the pump power, from $\sim 1\%$ to $\sim 8\%$. To access the optical sideband response for detuning locking, we keep the VNA-based optical intput and output measurement and use a power splitter (PS, Mini-Circuits ZFRSC-123-S+, insertion loss $\sim 10$ dB) to combine the signal from the VNA and the SG.

To detect the optical-to-microwave conversion with the maximal measured efficiency of $\sim 10^{-5}$ with red-detuned pump $P_{\mathrm{pump}} \sim 3\,\mu\mathrm{W}$, we need the microwave crosstalk level to be lower than the estimated converted microwave signal power $P_{\mu} \sim 2\times10^{-17}$ W $\sim -136$ dBm. Meanwhile, we measure an RSA internal noise floor level of $\sim -155$ dBm/Hz and a microwave crosstalk $P_{\mathrm{c}} = 3.8\times10^{-16}$ W $\sim -124$ dBm with 25 dBm output from the SG to the EOM. The microwave crosstalk is determined to be mostly between the EOM and the on-chip electrodes and the RF probe that is in-touch with the electrodes. The crosstalk is not significant but is still much higher than the signal (Supplementary Figure 7(b)). To suppress the crosstalk, we utilize an electromagnetic interference (EMI) shielding that encloses all microwave instruments except the RSA. The EMI shielding brings the crosstalk level down to comparable or lower than the RSA noise floor (Supplementary Figure 7(b)).

We use a span of 50 Hz, a resolution bandwidth of $f_{\mathrm{bw}} = 1$ Hz and 10 averages on the RSA, corresponding to a noise floor of $\sim -155$ dBm/Hz and a sweep time of 50 sec for a single trace of power spectrum. The SG frequency and RSA center frequency $f_{\mu}$ are then swept simultaneously across 2 MHz around the peak conversion frequency. The power spectrum of the converted microwave signal is recorded at every frequency. The cavity-laser detuning $\Delta$ is verified and corrected before taking the next frequency point by the VNA optical sideband sweep. The total converted power is calculated by integrating the power spectral density within $f_{\mu} \pm 2f_{\mathrm{bw}}$ (Supplementary Figure 7(b)).

The peak conversion efficiency is calibrated similar to the method introduced in Sec. Supplementary Note 8. The optical sideband ratio $r = (7.5 \pm 0.2)\%$ is measured by the FFP-TF, and the converted microwave photon flux is calculated from the integrated converted microwave power. We have taken into account the non-negligible power in the first blue and red sidebands and neglected higher order sidebands when calculating the sideband photon flux from the total optical power. $n_{\mathrm{c}}$ is also calculated using the pump power when the modulation is active, which is $\sim 13\%$ smaller than the total optical power.

## Supplementary Note 10. THERMALLY INDUCED MECHANICAL RED SHIFT

During the conversion measurement with the measurement setup shown in the main text, the thermal mechanical noise spectrum is recorded at the same time with the RSA. A clear red shift of the OMC mechanical mode is observed and is shown in Supplementary Figure 8. Power spectral density (PSD) curves with different intracavity photon numbers at detuning $\Delta = -\omega_{\mathrm{m}}$ is plotted with different colors in linear scale. The extracted peak frequencies are shown on the right plot (blue). The expected optical stiffening from optomechanical backaction is also calculated for comparison (red) and is a minor effect comparing to the measured red shift. We attribute the red shift to thermal effect, where the thermal expansion caused by a higher temperature would increase device size and decrease the density, and lead to a lower mechanical mode frequency. The localized breathing mode and the mechanical waveguide modes have different overlaps with the heat profile generated by the optical mode and shift at different rates.

As a result, the OMC breathing mode not only slowly approaches the IDT center frequency, but also shifts faster and could go pass mechanical waveguide modes. We notice an asymmetric lineshape of the low power thermal mechanical PSD at low pump power as shown in Fig. 2(c) of the main text, where a waveguide mechanical mode frequency is

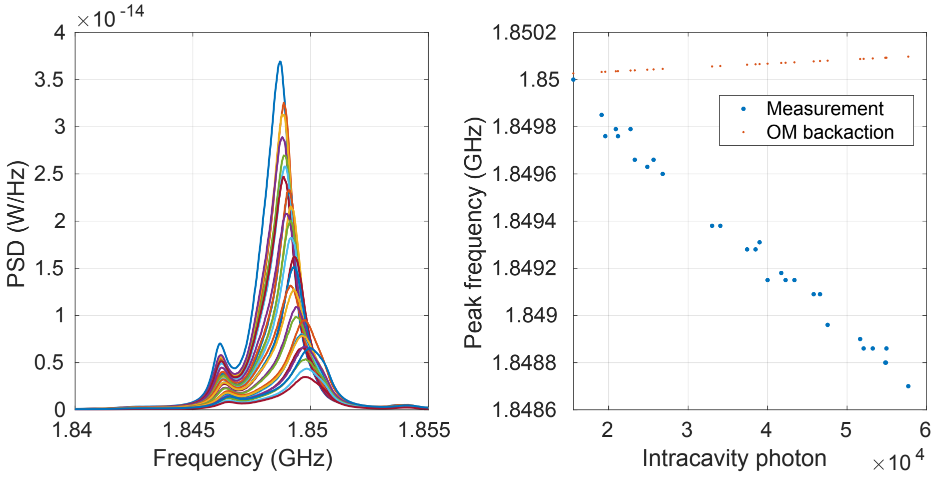


Supplementary Figure 8. Thermally induced mechanical frequency shift.

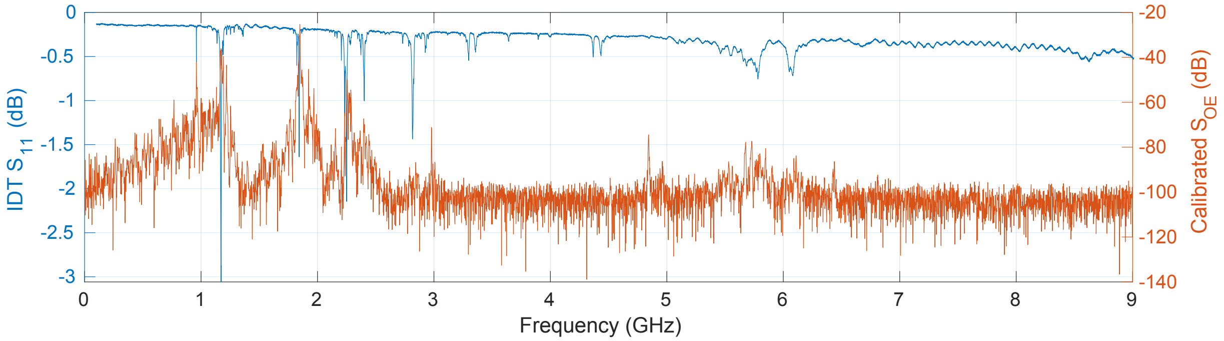


Supplementary Figure 9. Wide range data for IDT $S_{11}$ and microwave-to-optical $S$ parameter.

almost overlapping with the OMC breathing mode but is at a slightly lower frequency. In Supplementary Figure 8, the OMC breathing mode shifts across the same waveguide mode for increasing intracavity photon number from $n_\mathrm{c} \sim 2 \times 10^4$ to $n_\mathrm{c} \sim 4 \times 10^4$. The perfect overlap between the OMC breathing mode and a mechanical waveguide mode enhances the microwave-to-mechanical conversion efficiency and the total efficiency, which is clearly visible in Fig. 4(a) of the main text between $2 \times 10^4 < n_\mathrm{c} < 4 \times 10^4$.

## Supplementary Note 11. WIDE RANGE IDT RESPONSE AND MICROWAVE-TO-OPTICAL CONVERSION MEASUREMENT

We show the wide frequency range IDT $S_{11}$ response in Supplementary Figure 9. The IDT response is measured with identical setup as described in Ref. [5]. A typical microwave-to-optical $S_\mathrm{oe}$ parameter from 100 kHz to 9 GHz is also shown in Supplementary Figure 9. The $S_\mathrm{oe}$ parameter is shifted according to the calibrated peak efficiency.

For the microwave-to-optical $S$ parameter measurement, the VNA parameters used are (power, bandwidth, average, resolution) = (-50 dBm, 1 kHz, 1, 0.9 MHz). For the IDT $S_{11}$ measurement, the VNA parameters used are (power, bandwidth, average, resolution) = (-20 dBm, 10 kHz, 1, 10 kHz).

## SUPPLEMENTARY REFERENCES


[1] Safavi-Naeini, A. H. *et al.* Electromagnetically induced transparency and slow light with optomechanics. *Nature* **472**, 69 (2011).

[2] Jiang, W. *et al.* Lithium niobate piezo-optomechanical crystals. *Optica* **6**, 845–853 (2019).

[3] Hill, J. T., Safavi-Naeini, A. H., Chan, J. & Painter, O. Coherent optical wavelength conversion via cavity optomechanics. *Nature Communications* **3**, 1196–7 (2012).

[4] Fang, K., Matheny, M. H., Luan, X. & Painter, O. Optical transduction and routing of microwave phonons in cavity-optomechanical circuits. *Nature Photonics* **10**, 489–496 (2016).

[5] Dahmani, Y. D., Sarabalis, C. J., Jiang, W., Mayor, F. M. & Safavi-Naeini, A. H. Piezoelectric transduction of a wavelength-scale mechanical waveguide. *arXiv preprint arXiv:1907.13058* (2019).

[6] Alegre, T. P. M., Safavi-Naeini, A., Winger, M. & Painter, O. Quasi-two-dimensional optomechanical crystals with a complete phononic bandgap. *Optics Express* **19**, 5658–5669 (2011).

[7] MacCabe, G. S. *et al.* Phononic bandgap nano-acoustic cavity with ultralong phonon lifetime. *arXiv preprint arXiv:1901.04129* (2019).

[8] Chan, J. *Laser cooling of an optomechanical crystal resonator to its quantum ground state of motion*. Ph.D. thesis, California Institute of Technology (2012).

[9] Safavi-Naeini, A. H., Van Thourhout, D., Baets, R. & Van Laer, R. Controlling phonons and photons at the wavelength scale: integrated photonics meets integrated phononics. *Optica* **6**, 213–232 (2019).

[10] Clerk, A. A., Devoret, M. H., Girvin, S. M., Marquardt, F. & Schoelkopf, R. J. Introduction to quantum noise, measurement, and amplification. *Reviews of Modern Physics* **82**, 1155–1208 (2010).

[11] Helstrom, C. W. Quantum detection and estimation theory. *Journal of Statistical Physics* **1**, 231–252 (1969).

[12] Wiseman, H. M. & Milburn, G. J. *Quantum measurement and control* (Cambridge university press, 2009).

[13] Patel, R. N. *et al.* Single-mode phononic wire. *Physical Review Letters* **121**, 040501–6 (2018).
