# Supplementary material for: Efficient bidirectional piezo-optomechanical transduction between microwave and optical frequency
Source: Nat Commun. 2020 Mar 3;11:1166. doi: 10.1038/s41467-020-14863-3 (PMC7054291; doi:10.1038/s41467-020-14863-3)
Supplement: Supplementary file 2 — Peer Review File [file 41467_2020_14863_MOESM2_ESM.pdf]

**Editorial Note:** This manuscript has been previously reviewed at another journal that is not operating a transparent peer review scheme. This document only contains reviewer comments and rebuttal letters for versions considered at Nature Communications.

REVIEWERS' COMMENTS:

Reviewer #1 (Remarks to the Author):

The paper is good and a perfect match to NComms.

Reviewer #3 (Remarks to the Author):

The authors carefully responded to all the reviewers comments and I think the manuscript is in a great shape.

I have no further comments but would like to congratulate the authors for such a great and important work and strongly support publication in Nature Communications.
